# A call for phylogenetic context to understand geographic variation and host specificity in the parasitic copepod genus *Salmincola*

**DOI:** 10.1017/S0031182025100954

**Published:** 2025-11

**Authors:** Jeremy R. Abels, Jesse N. Weber

**Affiliations:** Department of Integrative Biology, University of Wisconsin-Madison, Madison, WI, USA

**Keywords:** copepods, host specificity, *Salmincola*, salmonids, taxonomy

## Abstract

Freshwater parasitic copepods appear to exhibit great taxonomic diversity. However, little is known about gene flow between species or whether there is incongruence between morphological and phylogenetic species definitions. Additionally, little is known about what evolutionary factors may contribute to speciation across various lineages. The copepod genus *Salmincola*, which includes common ectoparasites of fishes in the family Salmonidae, is distributed throughout the northern hemisphere and is a good model to demonstrate limited taxonomic understanding. Much of the regular scholarly output regarding *Salmincola* copepods comes from fisheries management agencies, where they are considered a pest species. Within a geographic region, *Salmincola* copepods of the same species are often found infecting their hosts at substantially different rates across different water bodies. However, present taxonomic definitions of *Salmincola* are based on decades old morphological descriptions, which were limited in geographic scope and number of specimens examined. There is a strong possibility that traditional species definitions in this genus, based on host species along with morphology, are missing cryptic diversity that may explain differences in infection intensity across environments. This review outlines the current scientific limitations of understanding of this genus and provides suggestions for how adding genetic data could inform taxonomic revisions, as well as clarifying connections between genetic differentiation and infection dynamics across localities.

## Graphical abstract

**Figure:**
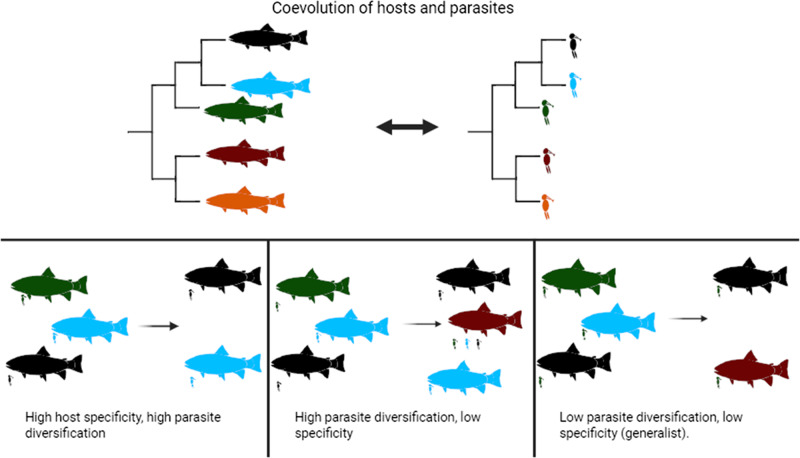

## Introduction

**Figure 1.**
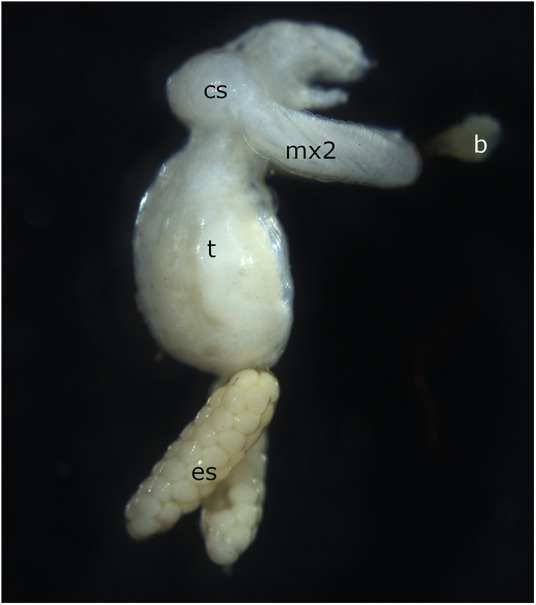
Adult female *Salmincola edwardsii* collected on brook trout near Richland Center, Wisconsin, USA. b, bulla; cs, cephalothorax; es, egg sac; mx2, second maxilla; t, trunk.

The Copepoda are a class of exceptionally diverse crustaceans, with nearly 3000 of the 13 000 estimated species being found in freshwater (Boxshall and Defaye, 2008). Around 330 species of freshwater copepods are parasitic, with fish being the most common host (Boxshall and Defaye, 2008). *Salmincola* is a genus of ectoparasitic copepods in the family Lernaeopodidae, which specializes on fishes in the family Salmonidae. *Salmincola* copepods begin life as free-swimming copepodid larvae and eventually attach themselves to the bodies of their host (Friend, 1942; Kabata, 1969). Host attachment is achieved via a structure called the bulla, which is secreted from the maxillae (Figure 1). Attachment sites vary across species. The most common attachment sites are the gills and fins, with the operculum, mouth and skin also being common (Kabata, 1969). *Salmincola* copepods are found throughout the northern hemisphere (Kabata, 1969), but beyond basic life history and geographical details, little is known about their growth and development. Moreover, a paucity of genomic studies leaves many open questions about the evolutionary history and radiation of the genus. *Salmincola* was first described to include 13 species (Wilson, 1915). A major revision of the genus was done by Kabata (1969), which increased the number of species to 15. At present, *Salmincola* has been found infecting a number of commercially and recreationally important species, including rainbow trout (*Oncorhynchus mykiss*), Brook trout (*Salvelinus fontinalis*) and Atlantic salmon (*Salmo salar*). For this reason, a large portion of the literature on this genus comes from fisheries management. Three new species have been described since 1970, all of which are found in Russia and Japan, where a large proportion of the evolutionary work on *Salmincola* has been done in recent decades. Additionally, only limited phylogenetic data, based on a handful of genes, exists for the genus (Hasegawa et al., 2022b; Shedko et al., 2023). A search of publicly available nucleotide sequence databases uncovered a total of 246 gene sequences across *Salmincola* (see Supplementary Table S1 for a summary of databases searched and available sequences). Although this may at first appear to be a reasonably large dataset, it only covers 3 genes that are primarily reported in 6 species.

Despite a long history of study in fisheries, significant gaps remain in the understanding of the evolutionary history of *Salmincola* copepods and their relationship with salmonid hosts. This review seeks to describe the current state of knowledge regarding the genus, with particular emphasis on species diversity and host specificity.

## Taxonomic review and natural history of the genus *Salmincola*

A literature search revealed 413 original records where authors provided genus and species for both host and parasite (see Supplementary Material 1 for a full list of references). Literature searches were conducted using Google Scholar. Search terms included ‘*Salmincola*’, ‘*Salmincola* copepod’ and ‘gill lice’. Further searches were done for each of the 23 described *Salmincola* species. In addition to English, searches for sources were conducted in Russian, Japanese, French, Ukrainian, Norwegian and Polish.

The complete list of records is summarized in Table 1, with records and references included in Supplementary Table S2. Reports from records before 1969 are revised to reflect current valid species names, based on Kabata (1969). In total, of the 23 described *Salmincola* species, 13 were rarely reported in the literature, suggesting that additional work is needed to determine if these are valid identifiers or merit scrutiny. Rare records in this case are defined as species with fewer than 10 validated recordings in the literature, regardless of host species. Rare occurrences of a parasite species on a given host genus were documented as well. Fifteen cases were identified where fewer than 10% of total records for a parasite species are related to a second host genus (highlighted in Table 1). Finally, an examination of broad geographic ranges of particular parasite species with abundant records was conducted. Given that species within salmonid genera tend to be closely related (further explanation provided later), hosts are generally discussed at the genus level in order to refrain from identifying potentially spurious pairings at the host species level.

**Table 1.** Host-parasite pairs found in the literature for *Salmincola*

*Salmincola* species	Host records *(number of papers/records)*
*Salmincola californiensis*	*Oncorhynchus clarki (6)*
	*Oncorhynchus gorbuscha (1)*
	*Oncorhynchus keta (1)*
	*Oncorhynchus kisutch (4)*
	*Oncorhynchus masou (6)*
	*Oncorhynchus mykiss (25)*
	*Oncorhynchus nerka (20)*
	*Oncorhynchus tshawytscha (23)*
	Prosopium williamsoni (1)^*^
	Salvelinus leucomaenis (1)^*^
	Salvelinus malma (1)^*^
	Salvelinus namaycush (2)^*^
	Salvelinus pluvius (1)^*^
*Salmincola carpionis*	*Oncorhynchus masou (1)*
	*Oncorhynchus mykiss (4)*
	*Oncorhynchus nerka (2)*
	*Salvelinus albus (2)*
	*Salvelinus alpinus (13)*
	*Salvelinus boganidae (1)*
	*Salvelinus fontinalis (6)*
	*Salvelinus kronocius (2)*
	*Salvelinus leucomaenis (7)*
	*Salvelinus levanidovi (1)*
	*Salvelinus malma (8)*
	*Salvelinus schmidti (3)*
	*Salvelinus taimyricus (2)*
	*Salvelinus taranetzi (1)*
*Salmincola coregonorum*	*Coregonus clupeaformis (1)*
	*Coregonus fera (1)*
	*Coregonus lavaretus (2)*
	*Coregonus widegreni (1)*
*Salmincola corpulentus*	*Coregonus artedi (1)*
	*Coregonus hoyi (2)*
*Salmincola cottidarum*	*Cottus kessleri (1)*
	*Paracotus kessleri (1)*
	*Paracotus kneri (1)*
*Salmincola edwardsii*	Coregonus maraena (1)^*^
	Coregonus nasus (1)^*^
	Cottus cognatus (1)^*^
	Oncorhynchus mykiss (5)^*^
	Oncorhynchus nerka (3)^*^
	Prosopium cylindraceum (1)^*^
	Prosopium williamsoni (1)^*^
	Salmo trutta (1)^*^
	*Salvelinus albus (1)*
	*Salvelinus alpinus (36)*
	*Salvelinus boganidae (1)*
	*Salvelinus curilus (1)*
	*Salvelinus czerkii (1)*
	*Salvelinus elgyticus (1)*
	*Salvelinus fontinalis (43)*
	*Salvelinus kronocius (2)*
	*Salvelinus lepechini (1)*
	*Salvelinus leucomaenis (3)*
	*Salvelinus malma (6)*
	*Salvelinus namaycush (1)*
	*Salvelinus neiva (1)*
	*Salvelinus schmidti (1)*
	*Salvelinus taranetzi (1)*
	*Salvelinus umbla (1)*
	Thymallus arcticus (2)^*^
*Salmincola exsanguinata*	*Salvelinus fontinalis (1)*
*Salmincola extensus*	*Coregonus artedi (3)*
	*Coregonus autumnalis (1)*
	*Coregonus clupeaformis (2)*
	*Coregonus lavaretus (3)*
	*Coregonus migratorius (1)*
	*Coregonus peled (1)*
	*Coregonus sardinella (4)*
	*Coregonus widegreni (1)*
	Salvelinus namaycush (1)^*^
	Prosopium cylindraceum (1)^*^
	Salvelinus alpinus (1)^*^
*Salmincola extumescens*	*Coregonus artedi (5)*
	*Coregonus autumnalis (2)*
	*Coregonus clupeaformis (5)*
	*Coregonus hoyi (2)*
	*Coregonus lavaretus (2)*
	*Coregonus migratorius (1)*
	*Coregonus nasus (1)*
	*Coregonus nelsonii (1)*
	*Coregonus peled (1)*
	Salvelinus namaycush (1)^*^
	Salmo salar (1)^*^
*Salmincola heintzi*	*Salvelinus salvelinus (1)*
*Salmincola jacuticus*	*Coregonus cylindratus (1)*
*Salmincola lavaretus*	*Coregonus baunti (1)*
	*Coregonus bicaulensis (1)*
	*Coregonus migratorius (2)*
	*Coregonus nasus (1)*
	*Coregonus sardinella (1)*
*Salmincola longimanus*	*Thymallus brevirostris (1)*
*Salmincola lotae*	*Lota lota (4)*
*Salmincola markewitcshi*	Parahucho perryi (1)^*^
	*Salvelinus fontinalis (2)*
	*Salvelinus leucomaenis (11)*
*Salmincola mica*	*Prosopium cylindraceum (1)*
*Salmincola nordmanni*	*Stenodus leucichthys (3)*
*Salmincola salmoneus*	*Salmo salar (18)*
	*Salmo trutta (7)*
	Thymallus thymallus (1)^*^
*Salmincola siscowet*	*Salvelinus namaycush (6)*
*Salmincola stellata*	*Parahucho perryi (11)*
*Salmincola strigatus*	*Coregonus sardinella (1)*
*Salmincola svetlani*	*Thymallus baicalensis (1)*
	*Thymallus nigrescens (1)*
*Salmincola thymalli*	Coregonus lavaretus (1)^*^
	*Prosopium cylindraceum (3)*
	Salmo trutta (1)^*^
	*Salvelinus alpinus (3)*
	*Thymallus arcticus (3)*
	*Thymallus baicalensis (2)*
	*Thymallus nigrescens (1)*
	*Thymallus signifer (1)*
	*Thymallus thymallus (8)*
	*Thymallus vulgaris (2)*

Host species marked with an asterisk

(*) represent host-genera which account for less than 10% of records for a given parasite.

## Natural history of *Salmincola*

### Host diversity

**Figure 2.**
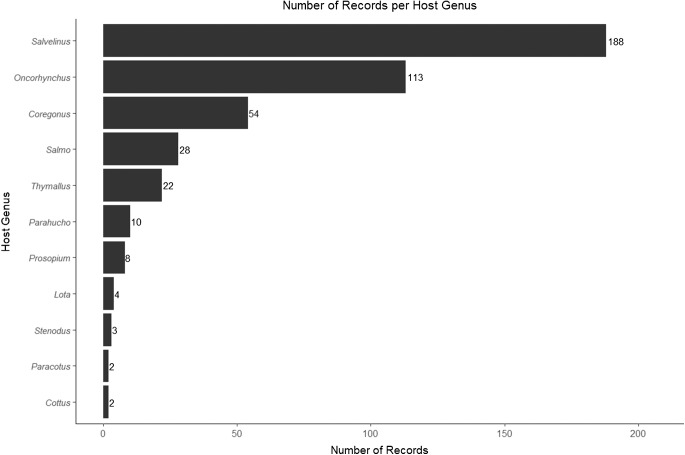
Bar chart depicting the frequency of *Salmincola* hosts.

*Salmincola* are known from all 4 subfamilies of Salmonidae; however, their presence across different species is highly variable between genera. Just 5 host genera, *Oncorhynchus, Salmo, Salvelinus, Coregonus* and *Thymallus*, collectively account for over 92% of records (Figure 2). As for the remaining records, there are 12 and 8 instances of infection in the salmonid genera *Prosopium* and *Parahucho*, respectively. Four records were found of *Salmincola lotae* infecting the burbot, *Lota lota*. Finally, 4 records were uncovered of host-family spillover into the family Cottidae, the sculpins. Except for 1 record of *S. edwardsii* infecting *Cottus cognatus*, all records of *Salmincola* infecting cottids are the species *S. cottidarum*. Further research is necessary to determine whether these records represent rare spillover events of other, more common *Salmincola* species or lineages unique to these non-salmonid hosts.

A central question for future research in *Salmincola* is the degree to which variation in host specificity correlates with morphological and genetic diversity in *Salmincola*. It is currently unclear, for instance, if *Salmincola* populations evolved alongside their host populations, how often parasite populations become extirpated, and how often new populations are established.

Species level definitions within Salmonidae are often debated, particularly in S*alvelinus, Coregonus* and *Thymallus.* A clear understanding of host species lineages will be key to understanding *Salmincola* diversity. Within *Salvelinus*, brook trout (*S. fontinalis*) is strongly supported and recognized as a valid species (Page and Burr, 2011). Dolly Varden trout (*S. malma*), Lake Trout (*S. namaycush*) and arctic charr (*S. alpinus*) are also well supported. Beyond those 4 lineages, most species of *Salvelinus* are extremely geographically constrained (Taylor, 2016; Osinov et al., 2021). *Coregonus*, the whitefishes, have a circumpolar distribution (Nelson et al., 2016). In North America, the cisco (*Coregonus artedi*) and the bloater (*Coregonus hoyi*) are well supported (Page and Burr, 2011). In Eurasia, the European whitefish (*Coregonus lavaretus*) is widely distributed but with significant morphological variation that is often interpreted as species diversity (Østbye et al., 2005; Bochkarev et al., 2017). In *Thymallus*, the number of extant, valid species ranges from just 2 (Gum et al., 2009) to 4 (Nelson et al., 2016) to 14 (Froese and Pauly, 2013). A consistent pattern across these 3 genera is a broad geographic distribution at the genus level with each region containing several well-supported species. Additionally, each genus contains a number of rarely reported species that are not nearly as strongly supported in the literature. In general, the species boundaries are less well documented in the remote regions of northeast Asia. While there may be an important relationship between host and parasite species diversity, further research will likely be hampered by disagreement over which evolutionary units, for both hosts and parasites, can be considered unique species. Importantly, jointly studying both hosts and parasites may help resolve some of these disputes. Testing for strong patterns of parasite specificity that support particular host species or clades could provide a novel approach to resolving several long-standing debates about Salmonid taxonomy.

### *Salmincola* diversity

**Figure 3.**
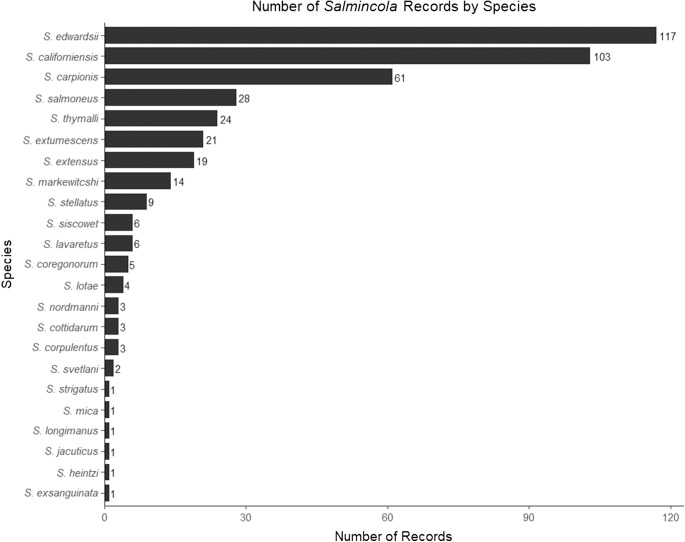
Bar chart showing frequency of records for 23 species of *Salmincola.*

The literature review identified large discrepancies in the number of publications supporting different *Salmincola* species designations. Over half (50.4%) of *Salmincola* records pertain to just 2 species: *Salmincola edwardsii* and *Salmincola californiensis*. The next 7 most-commonly reported species account for just over 40% of total records (Figure 3). The remaining records consist of 14 species with fewer than 10 records each, including 6 species for which there is just 1 record. Among the species described in the following, there is a large variation in host range. Most species are found primarily in a single host genus, and many of the uncommon and rare parasites have only been observed on a small group of hosts. However, instances of parasites infecting atypical host genera are common. While it is possible that these atypical infections constitute host spillover events, many of them could result from uneven study effort across *Salmincola* species. The host range of parasites commonly increases as they become more well studied (Poulin and Mouillot, 2003). Alternatively, given the slight morphological differences between some species, unusual host records may reflect parasite misidentification. Additionally, these rare species, which have not been examined phylogenetically, should be considered prime targets for future phylogenetic research.

The following sections provide a brief description of each major species in alphabetical order, followed by brief descriptions of the minor species organized by author. The summaries focus on recorded geographic distributions and host communities, and instances were identified where species are particularly relevant to diversification trends outlined earlier.

### Salmincola californiensis

*Salmincola californiensis* is among the most common *Salmincola* species. *S. californiensis* records exist from the United States and Canada (Kabata, 1969), Russia (Kazachenko and Matrosova, 2020), and Japan (Nagasawa and Urawa, 2002; Hasegawa et al., 2025a, b). In North America, the parasite is primarily found near the Pacific Coast, where *Salmincola californiensis* primarily infects salmonids of the genus *Oncorhynchus* (Kabata, 1969). However, there are some records of its presence in charr (genus *Salvelinus*) (Nagasawa et al., 1987; Reeves, 2015; Kazachenko and Matrosova, 2020). Morphologically, it is distinguished by the characteristic shortness of its trunk, which is ‘enough to make the species quite easy to recognize from its general appearance’ (Kabata, 1969). Over 93% of reports for this species are confined to a single host genus, *Oncorhynchus*, suggesting a large degree of specialization. Reports of *S. californiensis* from charr and whitefish (genus *Prosopium)* may represent spillover events, and further investigation is necessary to determine whether these reports may be capturing stable populations of *S. californiensis* outside of *Oncorhynchus*. However, it is possible that populations of *S. californiensis* infecting different hosts form distinct populations, or that parasites genetically cluster into discrete geographic units. Phylogeographic analyses offer the most straightforward method for understanding host and geographic patterns for *Salmincola.* For example, the phylogeography of another *Oncorhynchus* parasite, the flatworm *Gyrodactylus*, was recently documented using genetic data (Leis et al., 2021). That study not only uncovered distinct geographic units but also traced the evolutionary origin of the parasite as it jumped from the family Cyprinidae into salmonids and across salmonid subfamilies. Similar studies will be useful in determining the evolutionary history and geographic structure within *Salmincola*.

### Salmincola carpionis

*Salmincola carpionis* has a circumpolar distribution, being found in Iceland, Greenland, North America, and Northeast Asia (Kabata, 1969). While morphologically similar to *S. salmoneus, S. carpionis* has a distinctly shaped cephalothorax along with a thin portion of the trunk where it connects to the cephalothorax (Kabata, 1969). It primarily infects *Salvelinus*, with some records in *Oncorhynchus* (Moles, 1982; Nagasawa et al., 1995). While carpionis is not as common as *S. edwardsii* or *S. californiensis*, it remains among the most commonly reported *Salmincola* species (Figure 2).

### Salmincola coregonorum

*Salmincola coregonorum* is known primarily from records in the former USSR (Monod and Vladykov, 1931; Kabata, 1969), with a single record from Canada (Chinniah and Threlfall, 1978).

Morphologically, it is similar to *S. thymalli*, but has a distinct bulla morphology (Kabata, 1969). Every recorded specimen of *S. coregonorum* was found infecting members of the genus *Coregonus* (Table 1). Given the paucity of records for this species, future work should aim to confirm whether *S. coregonorum* constitutes a distinct species.

### Salmincola corpulentus

*Salmincola corpulentus* is distributed in North America, from the Laurentian Great Lakes to the Great Slave Lake and Great Bear Lake in the north of Canada (Miller and Kennedy, 1948; Kabata, 1969; Chinniah and Threlfall, 1978). It is morphologically distinguished by the shape of the endopod of the second antenna (Kabata, 1988) along with the unique curvature of its egg sacs (Kabata, 1969; Bowen and Stedman, 1990). This species appears to infect bloaters (*Coregonus hoyi*) (Bowen and Stedman, 1990; Muzzall and Madejian, 2013) and cisco (*Coregonus artedi*) (Hoff et al., 1997). While this species is fairly commonly reported, future work should be done to compare it in more depth to other species found in the North American Great Lakes.

### Salmincola cottidarum

*Salmincola cottidarum* is known only from sporadic records from Lake Baikal (Kabata, 1969; Kabata and Koryakov, 1974). Unique to the genus *Salmincola*, it infects sculpins of the genera *Cottus* and *Paracottus*, rather than salmonids. Kabata (1969) described the species as being morphologically similar to *S. edwardsii*, though his description was based on just 3 individuals from a single locality. Given the uniqueness of this species’ host range, further investigation of sculpins as hosts of *Salmincola* is necessary.

### Salmincola edwardsii

*Salmincola edwardsii*, one of the most well-sampled of any species in the genus, has a wide circumpolar distribution. Morphologically, *S. edwardsii* is most easily identified by the characteristics of the rami on the second antenna (Kabata, 1969). Infections have been recorded in North America (Mitro, 2016), Japan (Hasegawa et al., 2022a), Norway (Refsnes, 2014), and far eastern Russia (Shedko et al., 2023). *S. edwardsii* primarily infects members of the genus *Salvelinus* (Table 1). This includes the widely distributed arctic charr (*S. alpinus*), Dolly Varden trout (*S. malma*) and brook trout (*S. fontinalis*), as well as many records from potentially obscure members of *Salvelinus* (Table 1). It should be noted that there is no clear consensus on the number of species within *Salvelinus*, with reputable sources ranging from just 3 valid species with wide geographic ranges to dozens of species with highly constrained ranges (Froese and Pauly, 2013; Taylor, 2016; Osinov et al., 2021) (Table 1). Within *Salvelinus*, there is a strong divide between species with large ranges (such as *Salvelinus fontinalis*) and those with very limited distribution (such as *Salvelinus neiva*). Given its large range and diverse host range, there are ample opportunities for further research into the genetic and morphological diversity of this species. For instance, testing for phylogenetic associations, like those which have been created for gill parasites of cichlids (Seidlová et al., 2022) and feather mites of warblers (Matthews et al., 2018), could be used to provide evidence for not only distinct lineages within S. *edwardsii* but also species-level classifications for *Salvelinus*.

### Salmincola exsanguinata

A single record exists for *Salmincola exsanguinata* (Sandeman and Pippy, 1967). The species was described as infecting brook trout (*Salvelinus fontinalis*) on the Avalon Peninsula in Newfoundland and was differentiated based on morphology. Given the paucity of records surrounding this species, further investigation of the diversity of parasites infecting brook trout in Newfoundland is needed to determine if *S. exsanguinata* is a valid species.

### Salmincola extensus

*Salmincola extensus* is distributed in the Great Lakes region of North America (Kabata, 1969; Leong and Holmes, 1981), and in Russia from the far east to as far west as the Ural Mountains (Kabata, 1969; Gavrilov et al., 2013; Gavrilov and Gos’kova, 2018). While not exceptionally common, *S. extensus* cannot be considered a rare species. Morphologically, this species has a much longer cephalothorax compared to other members of the genus (Kabata, 1969). Host records are primarily within the genus *Coregonus*, with single reports of a lake trout (*Salvelinus namaycush*) in Saskatchewan (Pietrock and Hursky, 2011), arctic charr in Alaska (*Salvelinus alpinus*) (West, 1986), and a round whitefish in Russia (*Prosopium cylindraceum*) (Boutorina and Busarova, 2023).

### Salmincola extumescens

*Salmincola extumescens* is found in both North America and northern Eurasia (Kabata, 1969). Morphologically, this species is distinguished by the shape of its second antenna (Kabata, 1969). Host records indicate that *S. extumescens* is nearly exclusive on *Coregonus*, with single records indicating a presence in *Salmo salar* and S*alvelinus namaycush* (Chinniah and Threlfall, 1978). Given the small number of reports from non-*Coregonus* species, it is unclear whether or not this morphological distinction is large enough to support identification in these non-standard hosts. Additionally, while this species is not particularly rare, it is notable as one of a group of species that seems to specialize on coregonins, including *S. extensus*. Future work should focus on comparing these species with one another and other local *Salmincola* species to examine whether the infection of *Coregonus* species evolved independently.

### Salmincola lotae

*S. lotae* exclusively infects burbot (*Lota lota*) (Table 1), but records for this species are sparse. While *S. lotae* was first identified in Russia and Finland, it now infects burbot in the Laurentian Great Lakes (Kabata, 1969). It is currently considered an invasive species in North America. However, the relative obscurity of this species means that its presence in the Great Lakes prior to its recent discovery in the 1930s cannot be ruled out.

Prior to any analysis on this species, its continued presence in burbot populations needs to be established. Phylogenetic clustering of North American *S. lotae* with other North American species rather than with European *S. lotae* would be a strong indicator of a spillover event rather than a recent invasion.

### Salmincola salmoneus

*S. salmoneus* has the most western distribution of any *Salmincola* species in Eurasia, being found in the British Isles (Kabata, 1969). This species is the only 1 to exclusively infect Atlantic salmon (*Salmo salar*) and brown trout (*Salmo trutta*). In North America, *S. salmoneus* is known to infect Atlantic salmon along the northeastern coast (Friend, 1942; Pippy, 1969; McGladdery and Johnston, 1988). *S. salmoneus* has been reported frequently and consistently infects members of the same genus.

Interestingly, there are no accounts of *S. salmoneus* infecting introduced brown trout in North America. Much of the historical movement of stocked fishes involves moving eggs rather than adult fish, providing an opportunity for introduced host populations to be free of parasites. However, brown trout co-occurs with Atlantic salmon in the eastern region of North America where *S. salmoneus* has been recorded (Page and Burr, 2011), providing an opportunity for cross-host infection. The reasons for this lack of cross-host infection are unclear and require further study. Information about invasion events and their timing may help distinguish recent range expansions of hosts and parasites.

### Salmincola thymalli

*Salmincola thymalli* is distributed throughout the northern hemisphere (Kabata, 1969). This species has been reported relatively frequently throughout its range. This is the only *Salmincola species* that specializes on grayling, predominantly the genus *Thymallus* (Kabata, 1969). *Thymallus* is distributed widely throughout the Palearctic and Nearctic, with there likely being less stocking influence on host genetics compared to *Oncorhynchus* and *Salvelinus* (Weiss et al., 2021). Given that host gene flow may have strong influences on parasite specialization, biogeographic variation of *S. thymalli* could provide an important contrast to other *Salmincola* species that infect hosts whose ranges have been dramatically affected by human movement and cultivation.

### Other *Salmincola* species

Kabata (1969) described *Salmincola jacuticus* as infecting *Coregonus*, but he also raised questions of whether it could be a synonym of *S. extensus.* Specifically, morphological variation between these species is primarily restricted to variation in size and proportion, rather than topology. While the number of mandibular teeth also, this trait can be variable within a single species. Given the lack of reports in the subsequent decades, it is likely that later records of *S. jacuticus*-like specimens were instead classified as *S. extensus. Salmincola nordmanni (*Kabata, 1969) is another species that, similar to *S. jacuticus*, is likely a synonym of *S. extensus*.

Another set of potentially rare species was identified by Burdukovskaya and Pronin (2010, 2016). *Salmincola lavaretus* was described as infecting *Coregonus spp.* in and around lake Baikal (Burdukovskaya and Pronin, 2010; Burdukovskaya and Pronin 2016; Dugarov et al., 2022). It is currently only known from Russia. *Salmincola longimanus was* collected from *Thymallus brevirostris* in Lake Baikal, while *S. svetlani* was collected from 2 *Thymallus* species in the same lake (Burdukovskaya and Pronin, 2010). Recent studies on the diversity of fish lineages in Lake Baikal (Sukhanova et al., 2012; Bogdanov and Knizhin, 2022) make these species interesting candidates for additional study, given the large diversity of salmonids in the area and the ancient age of Lake Baikal.

*Salmincola heintzi* was initially described as infecting *Salvelinus* in Russia (Monod and Vladykov, 1931). Kabata (1969) later described it as similar to *S. edwardsii*. Given the lack of records for nearly a century, it may be a synonym of *S. edwardsii.*

Shedko (2004) described *Salmincola mica* as a new species based on its unique morphology. This parasite infects the gills of the whitefish species *Prosopium cylindraceum* in the Anadyr River in the Chukchi Peninsula in eastern Russia and has not been reported since its initial description. *Salmincola markewitschi* was described from the Russian far east in 2002 (Shedko and Shedko, 2002) and is nearly exclusively found on members of the genus *Salvelinus*; only 1 specimen has been collected from taimen (*Parahucho perryi*) (Kazachenko and Matrosova, 2020). This species has also been documented extensively in Japan (Nagasawa, 2021; Nagasawa and Urawa, 2022; Hasegawa et al., 2022b). *Salmincola strigatus* was re-described by Kabata in 1969 as based on Markewitsch’s 1936 description. This species is exclusively known from taimen and has been reported extensively in Japan and Russia in recent decades (see supplemental materials for a full list of reports). *Salmincola strigatus* was originally described by Markewitsch in 1936. In Kabata’s (1969) revision of the genus, it was redescribed without further new specimens. The only subsequent report was in 2020, with *S. strigatus* infecting *Coregonus sardinella* in Russia (Nikulina and Polyaeva, 2020). As with other obscure members of *Salmincola*, the validity of *S. strigatus* is uncertain pending further study. *Salmincola siscowet* is distributed in North America and is only known to infect lake trout (*Salvelinus namaycush*) (Kabata, 1969). This species is morphologically similar to *S. edwardsii*.

## Future directions

Little is known about the genetic and morphological diversity of *Salmincola* populations on a global scale. While a large number of morphological studies exist, none integrate data from across a large portion of the genus’ range. Only a few studies have examined *Salmincola* variation using few genetic loci (Hasegawa et al., 2022b; Shedko et al., 2023); no studies have yet addressed genome-wide variation using next-generation sequencing (Supplemental Table S1). This lacuna is striking given the numerous areas where genetic analyses could clarify important evolutionary and ecological features of not only the parasites, but also their hosts. While ecological and species-specific features that could cloud taxonomic studies were previously identified, it is now highlighted why, where, and how further efforts would be most effective in contributing to the exploration of this fascinating group.

### Validity of current species boundaries

To date, the vast majority of species identifications and definitions in *Salmincola* are morphology-based. Many records originate from broad parasite screenings or fisheries management agencies. The degree to which independent morphological examinations took place in these studies varies greatly. In many cases, it must be called into question whether or not researchers had adequate knowledge of *Salmincola* morphology to make accurate species identifications. It is worthwhile considering particular cases where these approaches are most problematic. The common diagnostic anatomical traits for *Salmincola* include the shape of the maxilliped palps, the number of spines on the exopod, the ratio of the cephalothorax length to the bulla diameter, and the number of outgrowths on the maxilliped palps (Kabata, 1969; Nagasawa and Urawa, 2022). While all of these appear to be robust features, some authors have raised concerns about the validity of morphological definitions. Hasegawa et al. (2022b) found that *S. carpionis* and *S. markewitschi* were hard to identify morphologically due to high morphological variation in samples of *S. markewitschi* infecting whitespotted charr in Japan. All parasites in that study had the appropriate number of outgrowths on the maxilliped palps, consistent with the original description of *S. markewitschi* (Shedko and Shedko, 2002). Conversely, some specimens had no spines on the distal end of the exopod of the antenna and a small bulla diameter, traits more in line with the original description of *S. carpionis (*Kabata, 1969). Additionally, Hasegawa et al. (2022b) also found that 28S rDNA and COI sequences indicated these copepods form a single population in Japan. In addition to morphology, the attachment site of the parasite, which varies across species, has been used as an important discriminating trait for *Salmincola*. Intriguingly, there is some indication that species with broader host ranges may support a more diverse set of attachment sites. For example, *S. californiensis*, with many known hosts, can attach to the gills, operculum, fins and bodies of its hosts (Kabata, 1969). By contrast, *S. lotae*, with a single known host, is exclusively known from infections of the mouth cavity (Bagge and Hakkari, 1982; Lasee et al., 1988). However, given that there are hundreds of records for *S. californiensis* and just a handful for *S. lotae*, it may be possible that this pattern is an artefact of uneven study effort across *Salmincola*, similar to the influence of publication bias on host range (Poulin and Mouillot, 2003). These factors highlight the need for validating morphological identification with genetic data, especially when considering rare host-parasite species pairs.

In his foundational publication on *Salmincola*, Kabata (1969) defined species based on just a few samples. These morphological definitions still form the basis of anatomical identification today. Given the results of Hasegawa et al. (2022b), it could be the case that some lineages house a large degree of morphological variation, leading to misclassified new species from morphologically extreme individuals. There are no multiple-gene phylogenies of *Salmincola* to date, increasing the chance that published phylogenies may present inaccurate hypotheses. Critically, the use of only a single locus or a small number of loci may provide less accurate inferences about phylogenetic history compared to larger datasets (Maddison, 1997). Interestingly, even the most common species of *Salmincola* can be miscategorized by expert parasitologists. Kabata (1969) includes at least 1 case wherein a record of *S. edwardsii* was reexamined by the author and included as *S. californiensis*. If even those researchers with a strong background in identifying *Salmincola* morphology are uncertain about certain identifications, then this strongly argues for the need to incorporate species definitions via genetics. One possibility is that current morphology-based species definitions are too broad and may not accurately capture *Salmincola* diversity. Some *Salmincola* may have been evolving for millennia alongside their hosts, leading to substantial genetic divergence between geographically separated populations (Shedko et al., 2023). However, many morphological traits may remain unchanged for long periods of time simply due to stabilizing selection. This cryptic speciation could also lead to morphological convergence, where a number of populations with nearly identical morphological characteristics do not descend from the same common ancestor.

**Figure 4.**
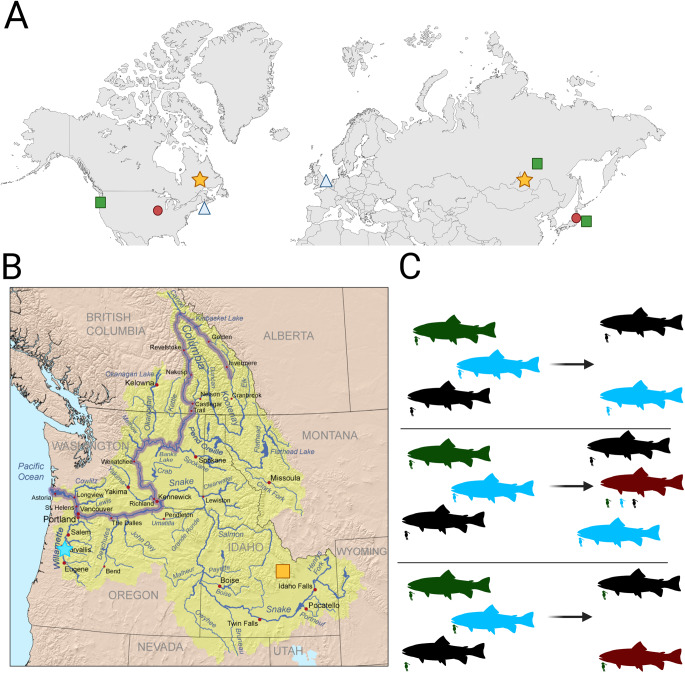
(A) Map of the northern hemisphere demonstrating localities of *Salmincola* populations representing species of particular interest to further study. Red circles: *Salmincola edwardsii* in Wisconsin and Japan. Blue triangles: *Salmincola salmoneus* in northeastern North America and northwestern Europe. Green squares: *Salmincola californiensis* along the western coast of North America and Japan. Gold stars: *Salmincola extumescens* in Newfoundland and around Lake Baikal. (B) Map of the Columbia River basin in western North America. Blue star: Willamette River, home to a diverse assemblage of *Oncorhynchus* populations known to be infected with *S. Californiensis*. Gold box: Birch Creek, home to a population of *O. Mykiss* which have only recently been reported to be infected with *S. Californiensis*. (B) Diagram depicting 3 possibilities for host specificity in *Salmincola*. Top: High host specificity, high parasite diversification. Middle: High parasite diversification, low specificity. Bottom: Low parasite diversification, low specificity (generalist).

The first genetic phylogeny of *Salmincola* was based on the COI gene and included 5 species (Shedko et al., 2023). The samples included in that study were primarily sourced from around far eastern Russia and Japan, with a few samples of *S. edwardsii, S. siscowet* and *S. californiensis* from North America. Interestingly, while the *S. californiensis* specimens grouped together by species, *S. edwardsii* from North America were more closely related to *S. siscowet* than they were to *S. edwardsii* from Asia. While this study anticipates the taxonomic improvements that could be made with genetic data, it also has important limitations. Most notably, population-level analyses based solely on mtDNA have serious limitations due to the lack of recombination in mitochondrial genomes (Rubinoff, 2006). Two methodological improvements would greatly aid future genetic work in this system; namely, greater number of loci and greater focus on particular species groups. In particular, a more in-depth study of *Salmincola edwardsii* and *Salmincola californiensis* from across continents is highly worth pursuing (Figure 4).

Some questions, such as genetic variation across geography, can be easily answered with relatively few loci, such as what is offered by approaches such as restriction-associated DNA-sequencing (Andrews et al., 2016). Multiple bioinformatic methods are suitable for inferring the extent of homogenization in Salmincola. Landscape genomics has become increasingly useful in delimiting species boundaries across geography (Chambers et al., 2025). Within *Salmincola edwardsii*, Cophylogenetic associations, like those which have been created for gill parasites of cichlids (Seidlová et al., 2022) and feather mites of warblers (Matthews et al., 2018), could be used to provide evidence for not only distinct lineages within S. *edwardsii* but also species-level classifications for *Salvelinus*. Utilization of these methods would help us to understand whether or not *Salmincola* edwardsii is best divided into multiple species, or whether *S. siscowet* is not diverged enough to be considered its own species distinct from *S. edwardsii*.

Although genetic data offer many avenues for future research, there is additional benefit to be gained from pairing these with larger-scale morphological datasets. The morphological variability noted in Hasegawa et al. (2022b) suggests that there may be significant morphological variation within *Salmincola*. This suggests that there is a substantial opportunity for morphological analyses of large numbers of *Salmincola* individuals. By focusing on the species mentioned earlier, *S. californiensis* and *S. edwardsii*, it might be possible to quite quickly collect a very large number of individuals, as these are the 2 most commonly reported species of *Salmincola* (Table 1). From there, it would be possible to develop a set of standardized landmarks for morphometric analysis. These analyses could then be paired with the genetic data collected for these species. This would allow for tests of whether the genomic and morphological data agree. By incorporating morphometrics, morphological variation could be much more easily quantified in *Salmincola*. Future work could then incorporate less common species using the same morphometrics. This would allow for a more complete accounting of the morphospace occupied by the genus *Salmincola* and inform species boundaries.

### Host specificity

There are substantial questions regarding the degree to which relationships between *Salmincola* copepods and their hosts are highly specialized (i.e. 1-to-1 species matching) or more general. To start, there is great variation across *Salmincola* species in the reported degree of host specialization. *S. lotae* and *S. siscowet* are each known to infect a single host, while *S. edwardsii* and *S. californiensis* parasitize many hosts. Some of this variation in host specificity may be determined by patterns of geographic isolation. Parasites with larger ranges tend to have more diverse host ranges (Krasnov et al., 2005). *Salmincola* species exhibit 3 distinct geographic distributions (Kabata, 1969). These include circumpolar, bicontinental and continental. There are indications that the pattern of more widely distributed species having a larger assemblage of hosts may hold true within *Salmincola*. For example, *S. edwardsii*, a species with a circumpolar distribution, is recorded in 25 host species (Table 1). *Salmincola siscowet*, however, is a continental species that is only known to infect 1 host, lake trout.

There are several plausible hypotheses concerning host specificity in *Salmincola* populations (Figure 4C). Given the wide scope of host range specificity in *Salmincola* (Table 1), no single hypothesis can account for every *Salmincola* species. Some species may exhibit complete specificity, in which each parasite species exhibits a strict association with a species or group of species, showing little or no evidence of host switching. Another hypothesis suggests partial specificity, whereby parasite populations may broadly track the evolutionary divergence of their hosts (cophylogenetic variation; see Paterson and Banks, 2001), yet retain the capacity to infect novel hosts, indicating incomplete host fidelity. Finally, there is generalism, where *Salmincola* species are capable of infecting a wide range of host species, including those they have not previously encountered. Genomic and morphometric studies will be able to determine which of these theoretical frameworks is most reflective of reality.

*Salmincola californiensis* is a promising candidate species for examining the validity of these frameworks, due to its high host diversity within the genus *Oncorhynchus* (Table 1). Even within the same river system, a single population of *S. californiensis* may infect multiple host species, as is the case in the Willamette River system in Oregon (Figure 4). These species include *Oncorhynchus tshawytscha* (Beeman et al., 2015; Monzyk et al., 2015; Herron-Seeley, 2016; Herron et al., 2018, 2024)*, Oncorhynchus clarki* (Monzyk et al., 2015)*, Oncorhynchus nerka* (Monzyk et al., 2015) *and Oncorhynchus mykiss* (Roon, 2014; Monzyk et al., 2015). By studying these *Salmincola* populations, it may be possible to better understand host specificity within a single species of *Salmincola*. When considering all populations of *S. californiensis*, host specificity for that species within *Oncorhynchus* will be most similar to the ‘generalist’ hypothesis. However, localized host specificity may still occur within populations. A single parasite species may specialize on a local host or host, while the entire species may remain a generalist with a large host range (Poulin et al., 2011). Further effort should also be directed to understanding what signals are utilized by *Salmincola* parasites to detect suitable hosts. Finally, any hypotheses regarding differences in the degree of host specificity across *Salmincola* species and populations are likely to be influenced by the large disparity in historical study effort across *Salmincola*.

While most current research on *Salmincola* relies on naturally collected samples, more mechanistic questions could be advanced via laboratory studies. Previous work has examined infection rates of *Salmincola californiensis* on rainbow trout in the laboratory (Neal et al., 2021). This study demonstrated that infection rates are dependent on temperature and copepodid density in the laboratory. Other studies utilizing brook trout infected with *S. edwardsii* found that infection rates are also affected by host size and behaviour (Poulin et al., 1991a, 1991b). In contrast, field studies have indicated no relationship between temperature and *Salmincola* infection rates (Henriksen et al., 2023). These experiments could be expanded to include additional *Salmincola* and salmonid species, including experimental infections of nonstandard species pairs. For example, attempting experimental infections of *Oncorhynchus* species with *S. edwardsii* and *Salvelinus fontinalis* with *S. californiensis.* These experiments could address whether infection is less likely in non-standard species pairs and whether infection rate is also influenced by temperature and copepodid density in novel contexts.

### Range expansions

The close relationship between humans and salmonid fishes can act as both an impediment and an opportunity when considering genetic patterns within and across *Salmincola* species. For example, extensive stocking of rainbow trout (*Oncorhynchus mykiss)* has resulted in hybridization and the diminishing of unique genetic signals in many lineages of native trout (Consuegra et al., 2011; Yau and Taylor, 2013). Although rainbow trout have become a classic example of a hybrid swarm, this pattern holds for a number of stocked salmonids, including *Salmo salar* (*Salmincola salmoneus*) and *Salvelinus fontinalis* (*Salmincola edwardsii*). It is an open question whether similar patterns of admixture occur within *Salmincola* populations living on these stocked hosts. Admixture is a major confounding issue for evolutionary biologists studying *Salmincola* as it obscures natural genomic signatures of gene flow. However, these host-parasite pairs offer powerful opportunities to observe repeated, natural experiments.

Several species, populations, and localities hold particular promise for understanding whether *Salmincola* are experiencing similar genetic homogenization as their hosts (Figure 4). For example, populations of *Salmincola californiensis* are frequently reported from new localities where they were previously not known to occur (Figure 4) (Suchomel and Billman, 2021; Swain‐Menzel and Billman, 2023). *Salmincola californiensis* has also been found infecting farmed rainbow trout far to the east of the host’s native range, including as far east as New Jersey and West Virginia (Hoffman, 1984; Sutherland and Wittrock, 1985). These populations are believed to be introduced via the movement of eggs and adult fish (Hoffman, 1984). Future work should focus on documenting genetic and morphological variation within rainbow trout-infecting *S. californiensis* across the broad range of that host-parasite pair and comparing that variation to that seen across host species in an environment with an abundance of This will allow for a better understanding of what pattern of diversification (Figure 4) is most accurate for this species, and whether or not distinct, host-specific clades exist.

In addition to basic taxonomic and evolutionary questions, further study of *Salmincola* could help answer a number of applied fisheries management questions. For instance, infection levels may provide information about habitat quality and the general health of fish populations. To date, work on this question has been limited to brook trout and *S. edwardsii*. Habitat quality appears to influence the intensity of *Salmincola* infections in brook trout. Specifically, increases in temperature (Mitro et al., 2019) may reduce host body condition and increase opportunities for *Salmincola* infection. Regions at the southern limits of salmonid ranges may see overall lower host body condition and higher infection rates (Nagasawa, 2020; Hasegawa and Koizumi, 2024). Poor host body condition, which may occur downstream of habitat conditions, also led to higher rates of infection by *S. markewitschi* in whitespotted char in Japan (Hasegawa and Koizumi, 2023). An outbreak of *Salmincola edwardsii* in Wisconsin in 2012 was likely caused by unseasonably warm water temperatures (Mitro, 2016). Infection by *Salmincola* is associated with increased mortality (Neal et al., 2021) and decreased recruitment (Mitro, 2016). Because of these serious impacts of outbreaks on fish stocks, future studies should work to develop an environmental framework for understanding when and where *Salmincola* infection will be most intense. An improved understanding of the environmental factors underlying *Salmincola* infection will help managers preserve salmonid stocks.

## Conclusions

Despite over 100 years of study suggesting that *Salmincola* display great richness in species and host diversity, and that these parasites occupy a wide geographic range, much work remains to understand the diversity and evolution of this genus. *Salmincola* was first described in 1915, and the most recent major revision, from 1969, is now nearly 60 years out of date. Additionally, a number of rare species, such as *S. mica* and *S. cottidarum*, have been reported only sparsely in the literature and in many cases have not been reported in many years. Furthermore, the boundaries between a number of species, including *S. siscowet* and *S. edwardsii*, and *S. carpionis* and *S. markewitschi*, are now in question due to recent evidence (Hasegawa et al., 2022b; Shedko et al., 2023). For these reasons, it is now time for a new taxonomic revision of the genus considering advances in genomics, newly described *Salmincola* species, and an improved understanding of the genus’ range given the large number of new publications in *Salmincola* in the intervening years. Studies should also prioritize the extent to which species found on multiple continents, such as *S. californiensis* and *S. edwardsii*, vary genetically and morphologically across these localities. For example, do these species display morphological or genetic geographic clines within continental sub-ranges? Finally, while almost all previous studies document wild infections, laboratory or highly controlled studies will almost certainly be necessary to understand the magnitude and mechanics of host specialization in apparent generalists such as *S. californiensis*.

There are significant opportunities within this system to further the understanding of host-parasite coevolution alongside improving the resources available to fisheries agencies to manage *Salmincola* infections. One question of considerable importance is why certain waterbodies have a substantial *Salmincola* presence while others do not, even with similar fish communities. Environmental conditions seem to play a role (Hasegawa and Koizumi 2024); however, there is a strong possibility that heritable specificity between hosts and parasites may also be important (Mitro, 2016; Mitro and Griffin, 2018). Genetic data will be essential for answering these questions.

## Supporting information

Abels and Weber supplementary material 1 Abels and Weber supplementary material

Abels and Weber supplementary material 2 Abels and Weber supplementary material

## Supplementary material

The supplementary material for this article can be found at https://doi.org/10.1017/S0031182025100954.
