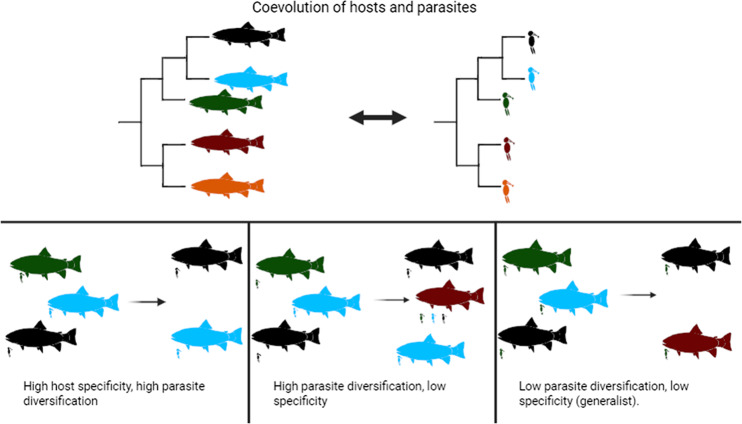# A call for phylogenetic context to understand geographic variation and host specificity in the parasitic copepod genus *Salmincola* - ERRATUM

**DOI:** 10.1017/S0031182025101145

**Published:** 2026-01

**Authors:** Jeremy R. Abels, Jesse N. Weber

This article originally published with the incorrect graphical abstract. This has now been corrected. The Publisher apologises for this error. The correct version is as follows:

**Figure:**