# Supplementary material for: A call for phylogenetic context to understand geographic variation and host specificity in the parasitic copepod genus Salmincola
Source: Parasitology. 2025 Oct 6;152(13):1299–311. doi: 10.1017/S0031182025100954 (PMC12917409; doi:10.1017/S0031182025100954)
Supplement: Abels and Weber supplementary material 1 — Abels and Weber supplementary material [file S0031182025100954sup001.docx]

**Adams, J. R.** (1956). A Parasitic Copepod (*Salmincola* falculata) Attached to a Fish Heart. *The Journal of Parasitology* **42**, 296. doi: [10.2307/3274856](https://doi.org/10.2307/3274856).

**Allison, L. N.** (1954). Advancements in Prevention and Treatment of Parasitic Diseases of Fish. *Transactions of the American Fisheries Society* **83**, 221–228. doi: [10.1577/1548-8659(1953)83[221:AIPATO]2.0.CO;2](https://doi.org/10.1577/1548-8659(1953)83%5B221:AIPATO%5D2.0.CO;2).

**Amundsen, P. ‐A., Kristoffersen, R., Knudsen, R. and Klemetsen, A.** (1997). Infection of *Salmincola edwardsii* (Copepoda: Lernaeopodidae) in an age‐structured population of Arctic charr—a long‐term study. *Journal of Fish Biology* **51**, 1033–1046. doi: [10.1111/j.1095-8649.1997.tb01542.x](https://doi.org/10.1111/j.1095-8649.1997.tb01542.x).

**Anderson, D. M.** (1993). Occurrence of *Salmincola siscowet* (Copepoda: Lernaeopodidae) on lake trout ( *Salvelinus namaycush* ) and lake trout backcross ( *S* . *namaycush* × *S* . *fontinalis* ) in Lake Huron. *Canadian Journal of Zoology* **71**, 2330–2333. doi: [10.1139/z93-327](https://doi.org/10.1139/z93-327).

**Bagge, P. and Hakkari, L.** (1982). The food and parasites of fish in some deep basins of northern L. Päijänne. In *Lakes and Water Management* (ed. Ilmavirta, V., Jones, R. I., and Persson, P.-E.), pp. 61–65. Springer Netherlands, Dordrecht doi: [10.1007/978-94-009-8003-7_8](https://doi.org/10.1007/978-94-009-8003-7_8).

**Bailey, R. E., Margolis, L. and Workman, G. D.** (1989). Survival of certain naturally acquired freshwater parasites of juvenile sockeye salmon, *Oncorhynchus nerka* (Walbaum), in hosts held in fresh and sea water, and implications for their use as population tags. *Canadian Journal of Zoology* **67**, 1757–1766. doi: [10.1139/z89-252](https://doi.org/10.1139/z89-252).

**Bailey, R. E.** (1981) A survey of the parasite fauna of juvenile Fraser River sockeye salmon, *Oncorhynchus nerka* (Walbaum), and the use of parasites in discriminating stocks. (Master thesis). Simon Fraser University, Vancouver, British Columbia, Canada. Available at: <https://core.ac.uk/download/pdf/56370575.pdf>

**Bailey, R. E. and Margolis, L.** (1987). Comparison of parasite fauna of juvenile sockeye salmon ( *Oncorhynchus nerka* ) from southern British Columbian and Washington State lakes. *Canadian Journal of Zoology* **65**, 420–431. doi: [10.1139/z87-063](https://doi.org/10.1139/z87-063).

**Bangham, R. V.** (1955). Studies on Fish Parasites of Lake Huron and Manitoulin Island. *American Midland Naturalist* **53**, 184. doi: [10.2307/2422308](https://doi.org/10.2307/2422308).

**Bangham, R. V. and Adams, J. R.** (1954). A Survey of the Parasites of Freshwater Fishes from the Mainland of British Columbia. *Journal of the Fisheries Research Board of Canada* **11**, 673–708. doi: [10.1139/f54-043](https://doi.org/10.1139/f54-043).

**Barndt, S. and Stone, J.** (2003). Infestation of *Salmincola californiensis* (Copepoda: Lernaeopodidae) in Wild Coho Salmon, Steelhead, and Coastal Cutthroat Trout Juveniles in a Small Columbia River Tributary. *Transactions of the American Fisheries Society* **132**, 1027–1032. doi: [10.1577/T02-122](https://doi.org/10.1577/T02-122).

**Beeman, J. W., Hansen, A. C. and Sprando, J. M.** (2015). Observational data on the effects of infection by the copepod *Salmincola californiensis* on the short- and long-term viability of juvenile Chinook salmon (*Oncorhynchus tshawytscha*) implanted with telemetry tags. *Animal Biotelemetry* **3**, 20. doi: [10.1186/s40317-015-0056-5](https://doi.org/10.1186/s40317-015-0056-5).

**Bell, G. R. and Margolis, L.** (1976). The Fish Health Program and the Occurrence of Fish Diseases in the Pacific Region of Canada. *Fish Pathology* **10**, 115–122. doi: [10.3147/jsfp.10.115](https://doi.org/10.3147/jsfp.10.115).

**Bennett, S. N., Adamson, M. L. and Margolis, L.** (1998). Long-term changes in parasites of sockeye salmon (*Oncorhynchus nerka*) smolts. **55**,.

**Bere, R.** (1930). THE PARASITIC COPEPODS OF THE FISH OF THE PASSAMAQUODDY REGION. *Contributions to Canadian Biology and Fisheries* **5**, 421–430. doi: [10.1139/f30-013](https://doi.org/10.1139/f30-013).

**Bergeron, M., Marcogliese, D. J. and Magnan, P.** (1997). The parasite fauna of brook trout, *Salvelinus fontinalis* (Mitchill), in relation to lake morphometrics and the introduction of creek chub, *Semotilus atromaculatus* (Mitchill). *Écoscience* **4**, 427–436. doi: [10.1080/11956860.1997.11682420](https://doi.org/10.1080/11956860.1997.11682420).

**Bergeron, M.** (1996). Impacts de l’introduction du mulet à cornes, Semotilus atromaculatus, et de la morphométrie des lacs sur la faune parasitaire de l’omble de fontaine, *Salvelinus fontinali*s, dans les lacs du Bouclier Laurentien. (Doctoral theis). Université du Québec à Trois-Rivières, Trois-Rivières, Québec, Canada. Available from: <https://depot-e.uqtr.ca/id/eprint/4740/1/000629063.pdf>

**Bertrand, M., Marcogliese, D. J. and Magnan, P.** (2008). Trophic polymorphism in brook charr revealed by diet, parasites and morphometrics. *Journal of Fish Biology* **72**, 555–572. doi: [10.1111/j.1095-8649.2007.01720.x](https://doi.org/10.1111/j.1095-8649.2007.01720.x).

**Bertrand, M.** (2004). Faune parasitaire, morphologie et contenus stomacaux comme indicateurs des habitats utilisés par l’omble de fontaine, *Salvelinus fontinalis*. (Master thesis). Université du Québec à Trois-Rivières, Trois-Rivières, Québec, Canada. Available from: <https://depot-e.uqtr.ca/id/eprint/4662/1/000109662.pdf>

**Beverley-Burton, M.** (1978). Metazoan parasites of arctic char ( *Salvelinus alpinus* L.) in a high arctic, landlocked lake in Canada. *Canadian Journal of Zoology* **56**, 365–368. doi: [10.1139/z78-052](https://doi.org/10.1139/z78-052).

**Black, G. A., Montgomery, W. L. and Whoriskey, F. G.** (1983). Abundance and distribution of *Salmincola edwardsii* (Copepoda) on anadromous brook trout, *Salvelinus fontinalis* , (Mitchill) in the Moisie River system, Quebec. *Journal of Fish Biology* **22**, 567–575. doi: [10.1111/j.1095-8649.1983.tb04216.x](https://doi.org/10.1111/j.1095-8649.1983.tb04216.x).

**Black, G. A.** (1981). Metazoan parasites as indicators of movements of anadromous brook charr ( *Salvelinus fontinalis* ) to sea. *Canadian Journal of Zoology* **59**, 1892–1896. doi: [10.1139/z81-257](https://doi.org/10.1139/z81-257).

**Bogdanov, B. E. and Knizhin, I. B.,** (2022). The Salmonid fishes of Lake Baikal and its adjacent water systems: annotated checklist with new taxa description. *Limnology and Freshwater Biology* 1688–1704. doi: [10.31951/2658-3518-2022-A-6-1688](https://doi.org/10.31951/2658-3518-2022-A-6-1688).

**Boutorina, T. E. and Busarova, О. Yu.** (2023). Таxonomical composition of fish parasites of the Penzhina and Talovka Rivers (the Sea of Okhotsk basin). *The researches of the aquatic biological resources of Kamchatka and the North-West Part of the Pacific Ocean* 55–69. doi: [10.15853/2072-8212.2023.69.55-69](https://doi.org/10.15853/2072-8212.2023.69.55-69).

**Bowen II, C. A. and Stedman, R. M.** (1990). Host–parasite relationships and geographic distribution of *Salmincola corpulentus* (Copepoda: Lernaeopodidae) on bloater ( *Coregonus hoyi* ) stocks in Lake Huron. *Canadian Journal of Zoology* **68**, 1988–1994. doi: [10.1139/z90-280](https://doi.org/10.1139/z90-280).

**Bowker, J. D., Carty, D. G., Wandelear, N., Schaffer, J., Swee, W. and LaPatra, S. E.** (2012). Efficacy of SLICE Premix (0.2% Emamectin Benzoate) for Reducing Infestations of *Salmincola* spp. on Freshwater‐Reared Rainbow Trout. *North American Journal of Aquaculture* **74**, 428–437. doi: [10.1080/15222055.2012.676019](https://doi.org/10.1080/15222055.2012.676019).

**Bristow, G. A.** (1993). Parasites of Norwegian freshwater salmonids and interactions with farmed salmon—a review. *Fisheries Research* **17**, 219–227. doi: [10.1016/0165-7836(93)90021-X](https://doi.org/10.1016/0165-7836(93)90021-X).

**Burdukovskaya, T. G. and Pronin, N. M.** (2010).Новые виды паразитических копепод рода *Salmincola* (Copepoda, Lernaeopodidae) из обонятельных ямок хариусовых (Thymallidae) и сиговых (Coregonidae) рыб бассейна оз. Bulletin of the Irkutsk State University. Series: Biology, Ecology **3**, 20-29.

**Burdukovskaya, T. G. and Pronin, N. M.** (2014). О стабильности многолетних показателей заражённости хариусовыхрыб озёр байкал (Россия) и хубсугул (Монголия) паразитическойкопеподой *Salmincola thymalli* (Copepoda: Lernaeopodidae). *Известия Иркутского государственного университета. Серия: Биология. Экология* **8**, 49–55.

**Burdukovskaya, T. G. and Pronin, N. M.** (2016). New host species and new distribution areas of *Salmincola lavaretus* (Copepoda: Lernaeopodidae): A parasite of coregonid fishes of Siberia. *Contemporary Problems of Ecology* **9**, 229–232. doi: [10.1134/S1995425516020025](https://doi.org/10.1134/S1995425516020025).

**Busarova, O. Y., Boutorina, T., Markevich, G. and Anisimova, L.** (2016). Parasite fauna of the landlocked sockeye salmon (*Oncorhynchus nerka*) of the Lake Kronotskoe (Kamchatka). *Parazitologiya* **50**, 211-223.

**Busarova, O. Yu., Esin, E. V. and Markevich, G. N.** (2022). Trophic Relations between Native *Salvelinus malma* Walb. and Introduced *Oncorhynchus nerka* Walb. in the Landlocked Lake Sevo, Kamchatka. *Inland Water Biology* **15**, 160–169. doi: [10.1134/S1995082922020031](https://doi.org/10.1134/S1995082922020031).

**Busarova, O. Yu., Koltun, G. G. and Podvalova, V. V.** (2018). Parasites of Salmonidae in the Azabachye Lake (Kamchatka), dangerous for human health or affecting quality and commercial value of fish. *The researches of the aquatic biological resources of Kamchatka and of the north-west part of the Pacific Ocean* **48**, 31–42. doi: [10.15853/2072-8212.2018.48.31-42](https://doi.org/10.15853/2072-8212.2018.48.31-42).

**Busarova, O. Yu., Markevich, G. N., Knudsen, R. and Esin, E. V.** (2017). Trophic differentiation of the nosed charr *Salvelinus schmidti* Viktorovsky, 1978 in Lake Kronotskoe (Kamchatka). *Russian Journal of Marine Biology* **43**, 57–64. doi: [10.1134/S1063074017010023](https://doi.org/10.1134/S1063074017010023).

**Butorina, T. E., Shed’ko, M. B. and Gorovaya, O. Yu.** (2008). Specific features of ecology of chars of the genus *Salvelinus* (Salmonidae) from the basin of Lake Kronotskoe (Kamchatka) according to parasitological data. *Journal of Ichthyology* **48**, 622–636. doi: [10.1134/S0032945208080080](https://doi.org/10.1134/S0032945208080080).

**Butorina, T. E., Shed’ko, M. B. and Gorovaya, O. Yu.** (2008). Specific features of ecology of chars of the genus *Salvelinus* (Salmonidae) from the basin of Lake Kronotskoe (Kamchatka) according to parasitological data. *Journal of Ichthyology* **48**, 622–636. doi: [10.1134/S0032945208080080](https://doi.org/10.1134/S0032945208080080).

**Buttner, J. K. and Hamilton, R. W.** (1976). *Ergasilus* (Copepoda: Cyclopoida) Infestation of Coho and Chinook Salmon in Lake Michigan. *Transactions of the American Fisheries Society* **105**, 491–493. doi: [10.1577/1548-8659(1976)105<491:ECIOCA>2.0.CO;2](https://doi.org/10.1577/1548-8659(1976)105%3C491:ECIOCA%3E2.0.CO;2).

**Buttner, J. K. and Heidinger, R. C.** (1979). First reported occurrence of *Salmincola californiensis* (Dana 1852)(Copepoda: Lernaeopodidae) east of the Rocky Mountains. *The Journal of parasitology* **65**, 320.

**Byrne, C. J., Grey, C., Holland, C. and Poole, R.** (2000). Parasite community similarity between four Irish lakes. *Journal of Helminthology* **74**, 301–305. doi: [10.1017/S0022149X00701064](https://doi.org/10.1017/S0022149X00701064).

**Byrne, C. J., Holland, C. and Tully, O.** (1999). Metazoan parasite community structure of sea trout on the west coast of Ireland. *Journal of Fish Biology* **55**, 127–134. doi: [10.1111/j.1095-8649.1999.tb00662.x](https://doi.org/10.1111/j.1095-8649.1999.tb00662.x).

**Byrne, C. J., Holland, C. V., Poole, R. and Kennedy, C. R.** (2002). Comparison of the macroparasite communities of wild and stocked brown trout ( *Salmo trutta* L.) in the west of Ireland. *Parasitology* **124**, 435–445. doi: [10.1017/S0031182001001330](https://doi.org/10.1017/S0031182001001330).

**C.P. Gallagher, T.A. Dick, J.A. Babaluk, and J.D. Reist** (2009). Communauté de parasites de l’omble chevalier, *Salvelinus alpinus*, du lac Hazen et du lac Craig, park national du Canada de Quttinirpaaq (Nunavut). doi: [10.13140/RG.2.1.1993.1365](https://doi.org/10.13140/RG.2.1.1993.1365).

**Chambers, E. A., Lara-Tufiño, J. D., Campillo-García, G., Cisneros-Bernal, A. Y., Dudek, D. J., León-Règagnon, V., Townsend, J. H., Flores-Villela, O. and Hillis, D. M.** (2025). Distinguishing species boundaries from geographic variation. *Proceedings of the National Academy of Sciences* **122**, e2423688122. doi: [10.1073/pnas.2423688122](https://doi.org/10.1073/pnas.2423688122).

**Chigbu, P.** (2001). Occurrence and Distribution of *Salmincola californiensis* (Copepoda: Lernaeopodidae) on Juvenile Sockeye Salmon ( *Oncorhynchus nerka* ) in Lake Washington. *Journal of Freshwater Ecology* **16**, 615–620. doi: [10.1080/02705060.2001.9663853](https://doi.org/10.1080/02705060.2001.9663853).

**Chinniah, V. C. and Threlfall, W.** (1978). Metazoan arasites of fish from the Smallwood Reservoir, Labrador, Canada. *Journal of Fish Biology* **13**, 203–213. doi: [10.1111/j.1095-8649.1978.tb03427.x](https://doi.org/10.1111/j.1095-8649.1978.tb03427.x).

**Cone, D. K. and Ryan, P. M.** (1984). Population sizes of metazoan parasites of brook trout (*Salvelinus fontinalis*) and Atlantic salmon (*Salmo salar*) in a small Newfoundland lake. *Canadian Journal of Zoology* **62**, 130–133. doi: [10.1139/z84-020](https://doi.org/10.1139/z84-020).

**Cone, D. K. and Ryan, P. M.** (1984). Population sizes of metazoan parasites of brook trout ( *Salvelinus fontinalis* ) and Atlantic salmon ( *Salmo salar* ) in a small Newfoundland lake. *Canadian Journal of Zoology* **62**, 130–133. doi: [10.1139/z84-020](https://doi.org/10.1139/z84-020).

**Conley, D.** (1994). 3.2.13 Gill Maggot Disease (Genus *Salmincola*).

**Conley, D. C. and Curtis, M. A.** (1993). Effects of temperature and photoperiod on the duration of hatching, swimming, and copepodid survival of the parasitic copepod *Salmincola edwardsii*. *Canadian Journal of Zoology* **71**, 972–976. doi: [10.1139/z93-128](https://doi.org/10.1139/z93-128).

**Conneely, J. J. and McCarthy, T. K.** (1984). The metazoan parasites of freshwater fishes in the Corrib catchment area, Ireland. *Journal of Fish Biology* **24**, 363–375. doi: [10.1111/j.1095-8649.1984.tb04809.x](https://doi.org/10.1111/j.1095-8649.1984.tb04809.x).

**Consuegra, S., Phillips, N., Gajardo, G. and De Leaniz, C. G.** (2011). Winning the invasion roulette: escapes from fish farms increase admixture and facilitate establishment of non‐native rainbow trout. *Evolutionary Applications* **4**, 660–671. doi: [10.1111/j.1752-4571.2011.00189.x](https://doi.org/10.1111/j.1752-4571.2011.00189.x).

**DesDevises, Y., Arthur, J. R. and Pellerin-Massicotte, J.** (2011). Parasites of Anadromous Arctic Char (*Salvelinus alpinus* L.) from Two Sites in Ungava Bay (Quebec, Canada). *JOURNAL-HELMINTHOLOGICAL SOCIETY WASHINGTON*, **65**, 87-90.

**Dick, T. A. and Belosevic, M.** (1981). Parasites of arctic charr *Salvelinus alpinus* (Linnaeus) and their use in separating sea‐run and non‐migrating charr. *Journal of Fish Biology* **18**, 339–347. doi: [10.1111/j.1095-8649.1981.tb03775.x](https://doi.org/10.1111/j.1095-8649.1981.tb03775.x).

**Dieterman, D. and Mitro, M.** (2019). Stream habitat needs for brook trout and brown trout in the Driftless Area. *Special Publication of the 11th Annual Driftless Area Symposium* pp. 29–44

**Dugarov, Z. N., Baldanova, D. R., Sondueva, L. D., Burdukovskaya, T. G., Khamnueva, T. R., Tolochko, L. V., Batueva, M. D.-D. and Zhepkholova, O. B.** (2022). Long-term dynamics of the parasitological situation in the Baikal omul in the Chivyrkuisky bay of Lake Baikal. *География и природные ресурсы* **43**, 170–178. doi: [10.15372/GIPR20220518](https://doi.org/10.15372/GIPR20220518).

**Farooqi, M., Nicholson, S., Aprahaminan, M. and Douglas, S.** (1992). *Arctic charr from Ennerdale Water: A pilot Study NRA Northwest Region Report nos*. NRA/NW/FTR/92/2.

**Fasten, N.** (1916). THE EYE OF THE PARASITIC COPEPOD, *SALMINCOLA EDWARDSII* OLSSON (LERNÆOPODA EDWARDSII OLSSON). *The Biological Bulletin* **31**, 407-[418]-1. doi: [10.2307/1536319](https://doi.org/10.2307/1536319).

**Fasten, N.** (1920). Morphology and attached stages of first copepodid larva of *Salmincola edwardsii*. *Publications* Puget Sound Biological Station **2**, 153.

**Fasten, N.** (1921a). ANOTHER MALE COPEPOD OF THE GENUS *SALMINCOLA* FROM THE GILLS OF THE CHINOOK SALMON. *The Biological Bulletin* **41**, 121-[124]-1. doi: [10.2307/1536743](https://doi.org/10.2307/1536743).

**Fasten, N.** (1921b). Studies on Parasitic Copepods of the Genus *Salmincola*. *The American Naturalist* **55**, 449–456. doi: [10.1086/279828](https://doi.org/10.1086/279828).

**Finley, M. B.** (1992). *A survey of parasites of selected fish from the Kuskokwim River Delta Region, Alaska*. (Master thesis). California Polytechnic State University. San Luis Obispo, California, USA. Available from: <https://search.proquest.com/openview/71abf94d2577814270c41f1dd1cf806c/1?pq-origsite=gscholar&cbl=18750&diss=y>

**Fjær, M. A. D.** (2019). Pukkellaks (*Oncorhynchus gorbuscha*) tatt på Vestlandet.-Hvilke parasitter og infeksjoner bærer de på? (Master Thesis). University of Bergen, Bergen, Norway. Available at: <https://bora.uib.no/bora-xmlui/bitstream/handle/1956/20403/Marte-Andrea-Fj-r-Masteroppgave-FISKEHELSE-JUNI-2019.pdf>

**Friend, G. F.** (1942). XV.—The Life-history and Ecology of the Salmon Gill-Maggot *Salmincola salmonea* (L.) (Copepod Crustacean). *Transactions of the Royal Society of Edinburgh* **60**, 503–541. doi: [10.1017/S008045680001797X](https://doi.org/10.1017/S008045680001797X).

**Frimeth, J. P.** (1987a). A survey of the parasites of nonanadromous and anadromous brook charr ( *Salvelinus fontinalis* ) in the Tabusintac River, New Brunswick, Canada. *Canadian Journal of Zoology* **65**, 1354–1362. doi: [10.1139/z87-215](https://doi.org/10.1139/z87-215).

**Frimeth, J. P.** (1987b). Potential use of certain parasites of brook charr ( *Salvelinus fontinalis* ) as biological indicators in the Tabusintac River, New Brunswick, Canada. *Canadian Journal of Zoology* **65**, 1989–1995. doi: [10.1139/z87-303](https://doi.org/10.1139/z87-303).

**Fryer, G.** (1966). Habitat Selection and Gregarious Behaviour in Parasitic Crustaceans. *Crustaceana* **10**, 199–209. doi: [10.1163/156854066X00720](https://doi.org/10.1163/156854066X00720).

**Fryer, G.** (1981). The copepod *Salmincola edwardsii* as a parasite of *Salvelinus alpinus* in Britain, and a consideration of the so‐called relict fauna of Ennerdale Water. *Journal of Zoology* **193**, 253–268. doi: [10.1111/j.1469-7998.1981.tb03443.x](https://doi.org/10.1111/j.1469-7998.1981.tb03443.x).

**Gaevskaja, A. V.** (1991). Pasożytnicze Crustacea ryb północno-wschodniego Atlantyku. *Wiadomości Parazytologiczne* **37**, 145-148.

**Gall, G. A. E., Mcclendon, E. L. and Schafer, W. E.** (1972). Evidence on the Influence of the Copepod (*Salmincola californiensis*) on the Reproductive Performance of a Domesticated Strain of Rainbow Trout (*Salmo gairdneri*). *Transactions of the American Fisheries Society* **101**, 345–346. doi: [10.1577/1548-8659(1972)101<345:EOTIOT>2.0.CO;2](https://doi.org/10.1577/1548-8659(1972)101%3C345:EOTIOT%3E2.0.CO;2).

**Gallagher, C. P. and Dick, T. A.** (2010). Trophic structure of a landlocked Arctic char *Salvelinus alpinus* population from southern Baffin Island, Canada. *Ecology of Freshwater Fish* **19**, 39–50. doi: [10.1111/j.1600-0633.2009.00387.x](https://doi.org/10.1111/j.1600-0633.2009.00387.x).

**Gavrilov, A. L., Bogdanov, V. D. and Ieshko, E. P.** (2013). Characteristics of parasitic infection in the least cisco, *Coregonus sardinella valenciennes*, 1848, in the Ural tributaries of the lower Ob. *Russian Journal of Ecology* **44**, 43–49. doi: [10.1134/S1067413613010062](https://doi.org/10.1134/S1067413613010062).

**Gavrilov, A. L. and Gos’kova, O. A.** (2018). The Long-Term Dynamics of Parasite Infection in Coregonids with Different Food Specializations. *Russian Journal of Ecology* **49**, 548–553. doi: [10.1134/S1067413618060073](https://doi.org/10.1134/S1067413618060073).

**Grammens, B. and College, C.** Incidence of *Salmincola californiensis* in Westslope Cutthroat Trout in Relation to Brook Trout Density in the Lolo Creek Drainage.

**Gunn, C., Carty, D., Walker, P. G., Colburn, P. A. and Bowker, J. D.** (2012). Pilot Field Trial to Evaluate SLICE (0.2% Emamectin Benzoate)–Medicated Feed to Reduce a Natural Infestation of *Salmincola californiensis* in Freshwater‐Reared Rainbow Trout. *North American Journal of Aquaculture* **74**, 424–427. doi: [10.1080/15222055.2012.676015](https://doi.org/10.1080/15222055.2012.676015).

**Gurney, R.** (1945). IX.— *Some notes on the development and classification of parasitic Copepoda*. *Annals and Magazine of Natural History* **12**, 121–127. doi: [10.1080/00222934508527494](https://doi.org/10.1080/00222934508527494).

**Gurney, R.** (1947). XLIV.— *Some remarks on the morphology of the Copepoda*. *Annals and Magazine of Natural History* **14**, 493–500. doi: [10.1080/00222934708654658](https://doi.org/10.1080/00222934708654658).

**Hammar, J.** (2000). Cannibals and parasites: conflicting regulators of bimodality in high latitude Arctic char, *Salvelinus alpinus*. *Oikos* **88**, 33–47. doi: [10.1034/j.1600-0706.2000.880105.x](https://doi.org/10.1034/j.1600-0706.2000.880105.x).

**Hance, D. J., Kock, T. J., Perry, R. W. and Pope, A. C.** (2022). *Assessing the efficacy of using a parentage-based tagging survival model to evaluate two sources of mortality for juvenile Chinook salmon (Oncorhynchus tshawytscha) in Lookout Point Reservoir, Oregon*, 2331–1258. US Geological Survey.

**Hanek, G. and Molnar, K.** (1974). Parasites of Freshwater and Anadromous Fishes from Matamek River System, Quebec. *Journal of the Fisheries Research Board of Canada* **31**, 1135–1139. doi: [10.1139/f74-129](https://doi.org/10.1139/f74-129).

**Hare, G. M. and Frantsi, C.** (1974). Abundance and Potential Pathology of Parasites Infecting Salmonids in Canadian Maritime Hatcheries. *Journal of the Fisheries Research Board of Canada* **31**, 1031–1036. doi: [10.1139/f74-116](https://doi.org/10.1139/f74-116).

**Hargis, L. N., Lepak, J. M., Vigil, E. M. and Gunn, C.** (2014). Prevalence and intensity of the parasitic copepod ( *Salmincola californiensis* ) on Kokanee salmon ( *Oncorhynchus nerka* ) in a reservoir in Colorado. *The Southwestern Naturalist* **59**, 126–129. doi: [10.1894/N06-JC-72.1](https://doi.org/10.1894/N06-JC-72.1).

**Hasegawa, R., Ayer, C. G., Umatani, Y., Miura, K., Ukumura, M., Katahira, H. and Koizumi, I.** (2022a). Potential negative effects and heterogeneous distribution of a parasitic copepod *Salmincola edwardsii* (Copepoda: Lernaeopodidae) on Southern Asian Dolly Varden *Salvelinus curilus* in Hokkaido, Japan. *Parasitology International* **87**, 102529. doi: [10.1016/j.parint.2021.102529](https://doi.org/10.1016/j.parint.2021.102529).

**Hasegawa, R., Katahira, H. and Koizumi, I.** (2022b). *Salmincola markewitschi* or *S. carpionis* (Copepoda: Lernaeopodidae)? A requirement for taxonomic revision due to their high morphological variations. *Folia Parasitologica* **69**, 025. doi: [10.14411/fp.2022.025](https://doi.org/10.14411/fp.2022.025).

**Hasegawa, R., Sugimoto, Y. and Koizumi, I.** (2025b). A new locality record of the ectoparasitic copepod *Salmincola californiensis* in Japan: A potential glacial relict population? *Crustacean Research* **54**, 1–7. doi: [10.18353/crustacea.54.0_1](https://doi.org/10.18353/crustacea.54.0_1).

**Hasegawa, R., Uemura, Y., Yamashita, Y., Inoshita, M. and Koizumi, I.** (2025a). Highly Threatened Status for the Relict Populations of Ectoparasitic Copepod *Salmincola californiensis* in Japan. *Aquatic Conservation: Marine and Freshwater Ecosystems* **35**, e70073. doi: [10.1002/aqc.70073](https://doi.org/10.1002/aqc.70073).

**Hasegawa, R. and Koizumi, I.** (2023). Parasites either reduce or increase host vulnerability to fishing: a case study of a parasitic copepod and its salmonid host. *The Science of Nature* **110**, 10. doi: [10.1007/s00114-023-01836-x](https://doi.org/10.1007/s00114-023-01836-x).

**Hasegawa, R. and Koizumi, I.** (2023). Parasites either reduce or increase host vulnerability to fishing: a case study of a parasitic copepod and its salmonid host. *The Science of Nature* **110**, 10. doi: [10.1007/s00114-023-01836-x](https://doi.org/10.1007/s00114-023-01836-x).

**Hasegawa, R. and Koizumi, I.** (2024). Consistent Negative Correlations between Parasite Infection and Host Body Condition Across Seasons Suggest Potential Harmful Impacts of *Salmincola markewitschi* on Wild White-Spotted Charr, *Salvelinus leucomaenis*. *Zoological Science* **41**,. doi: [10.2108/zs230028](https://doi.org/10.2108/zs230028).

**Hasegawa, R. and Koizumi, I.** (2024). Consistent Negative Correlations between Parasite Infection and Host Body Condition Across Seasons Suggest Potential Harmful Impacts of *Salmincola markewitschi* on Wild White-Spotted Charr, *Salvelinus leucomaenis*. *Zoological Science* **41**, 192-200. doi: [10.2108/zs230028](https://doi.org/10.2108/zs230028).

**Hassemer, P., Kline, P., Heindel, J., Plaster, K. and Venditti, D. A.** (2001). ANNUAL PROGRESS REPORT January 1, 1999 — December 31, 1999. Available from: <https://www.osti.gov/servlets/purl/819927>

**Henriksen, A.-C.** (2019) Seasonal and spatial variation in the macroparasite fauna of Arctic charr (*Salvelinus alpinus* L.) in Lake Norsjø, South-Eastern Norway. (Master thesis). University of South-eastern Norway, Kongsburg Norway. Available at: <https://openarchive.usn.no/usn-xmlui/bitstream/handle/11250/2611111/2019_Master_Henriksen.pdf?sequence=1>

**Henriksen, E. H., Frainer, A., Poulin, R., Knudsen, R. and Amundsen, P.** (2023a). Ectoparasites population dynamics are affected by host body size but not host density or water temperature in a 32‐year long time series. *Oikos* **2023**, e09328. doi: [10.1111/oik.09328](https://doi.org/10.1111/oik.09328).

**Henriksen, E. H., Frainer, A., Poulin, R., Knudsen, R. and Amundsen, P.** (2023b). Ectoparasite population dynamics are affected by host body size but not host density or water temperature in a 32‐year long time series. *Oikos* **2023**, e09328. doi: [10.1111/oik.09328](https://doi.org/10.1111/oik.09328).

**Herron, C. L., Kent, M. L. and Schreck, C. B.** (2018). Swimming Endurance in Juvenile Chinook Salmon Infected with *Salmincola californiensis*. *Journal of Aquatic Animal Health* **30**, 81–89. doi: [10.1002/aah.10010](https://doi.org/10.1002/aah.10010).

**Herron, C. L., Kent, M. L. and Schreck, C. B.** (2018). Swimming Endurance in Juvenile Chinook Salmon Infected with *Salmincola californiensis*. *Journal of Aquatic Animal Health* **30**, 81–89. doi: [10.1002/aah.10010](https://doi.org/10.1002/aah.10010).

**Herron, C. L., Ruse, N. E., Rockey, D. D., Sanders, J. L., Peterson, J. T., Schreck, C. B. and Kent, M. L.** (2024). *Aeromonas salmonicida* , causative agent of salmonid furunculosis, isolated from the freshwater parasitic copepod, *Salmincola californiensis*. *Journal of Fish Diseases* **47**, e13885. doi: [10.1111/jfd.13885](https://doi.org/10.1111/jfd.13885).

**Herron, C. L., Ruse, N. E., Rockey, D. D., Sanders, J. L., Peterson, J. T., Schreck, C. B. and Kent, M. L.** (2024). *Aeromonas salmonicida* , causative agent of salmonid furunculosis, isolated from the freshwater parasitic copepod, *Salmincola californiensis*. *Journal of Fish Diseases* **47**, e13885. doi: [10.1111/jfd.13885](https://doi.org/10.1111/jfd.13885).

**Herron-Seeley, C. H.** (2016). The Impact of Parasitic Copepod *Salmincola Californiensis* on Swimming Ability & Oxidative Burst Activity in Response to Stress in Juvenile Chinook Salmon. (Master Thesis). Oregon State University, Corvallis, Oregon, USA. Available at: <https://ir.library.oregonstate.edu/downloads/rx913t650>

**Hicks, F. J. and Threlfall, W.** (1973). Metazoan parasites of salmonids and coregonids from coastal Labrador*. *Journal of Fish Biology* **5**, 399–415. doi: [10.1111/j.1095-8649.1973.tb04468.x](https://doi.org/10.1111/j.1095-8649.1973.tb04468.x).

**Hiramatsu, N., Fukuda, H., Kitamura, M., Shimizu, M., Fuda, H., Kobayashi, K. and Hara, A.** (2001). Serum immunoglobulin M (IgM) in Sakhalin taimen (Hucho perryi) purification, characterization, circulating levels, and specific IgM production by the parasitic *Salmincola stellatus*. *Aquaculture Science* **49**, 347–355.

**Hoff, M. H., Pronin, N. M. and Baldanova, D. R.** (1997). Parasites of lake herring (*Coregonus arted*i) from Lake Superior, with special reference to use of parasites as markers of stock structure. *Journal of Great Lakes Research* **23**, 458–467.

**Hoffman, G. L.** (1984) *Salmincola californiensis* continues the march eastward. *American Fisheries Society Fish Health Section Newsletter* **12, 5-5** Available at: <https://units.fisheries.org/fhs/about/homepage-new/>

**Johnson, K. A. and Heindel, J. A.** (2001). Efficacy of manual removal and ivermectin gavage for control of *Salmincola californiensis* (Wilson) infestation of chinook salmon, *Oncorhynchus tshawytscha* (Walbaum), captive broodstocks. *Journal of Fish Diseases* **24**, 197–203. doi: [10.1046/j.1365-2761.2001.00279.x](https://doi.org/10.1046/j.1365-2761.2001.00279.x).

**Johnson, M., Tetzlaff, S., Katz, A. and Sperry, J.** (2024). Comparison of qPCR and metabarcoding for environmental DNA surveillance of a freshwater parasite. *Ecology and Evolution* **14**, e11382. doi: [10.1002/ece3.11382](https://doi.org/10.1002/ece3.11382).

**Johnston, C. E. and Dykeman, D.** (1987). Observations on body proportions and egg production in the female parasitic copepod ( *Salmincola salmoneus* ) from the gills of Atlantic salmon ( *Salmo salar* ) kelts exposed to different temperatures and photoperiods. *Canadian Journal of Zoology* **65**, 415–419. doi: [10.1139/z87-062](https://doi.org/10.1139/z87-062).

**Kabata, Z.** (1969). Revision of the Genus *Salmincola* Wilson, 1915 (Copepoda: Lernaeopodidae). *Journal of the Fisheries Research Board of Canada* **26**, 2987–3041. doi: [10.1139/f69-285](https://doi.org/10.1139/f69-285).

**Kabata, Z.** (1986). Redescriptions of and comments on four little-known Lernaeopodidae (Crustacea: Copepoda). *Canadian Journal of Zoology* **64**, 1852–1859. doi: [10.1139/z86-276](https://doi.org/10.1139/z86-276).

**Kabata, Z. and Cousens, B.** (1977). Host–Parasite Relationships Between Sockeye Salmon, *Oncorhynchus nerka* , and *Salmincola californiensis* (Copepoda: Lernaeopodidae). *Journal of the Fisheries Research Board of Canada* **34**, 191–202. doi: [10.1139/f77-029](https://doi.org/10.1139/f77-029).

**Kabata, Z. and Koryakov, E.** (1974). Морфологическая изменчивость *Salmincola cottidarum* Messjatzeff (Copepoda: Lernaeopodidae)-паразита бычков. *Паразитология.* **8**, 306.

**Kamerath, M., Allen, B. C. and Chandra, S.** (2009). First Documentation of *Salmincola californiensis* in Lake Tahoe, CA—NV, USA. *Western North American Naturalist* **69**, 257–259. doi: [10.3398/064.069.0216](https://doi.org/10.3398/064.069.0216).

**Kawanobe, M.** (2020). Hatching of *Salmincola carpionis* and period of parasitic capacity of the larvae. *Bulletin of Nagano Prefectural Fisheries Experimental Station (Japan)*.

**Kazachenko, V., and Matrosova, I. V.,**  (2020). Parasites (Hirudinea, Copepoda) of freshwater fishes of Primorsky Krai. Scientific works of Dalrybvtuza, **52**, 12-23.

**Kennedy, C. R.** (1974). A checklist of British and Irish freshwater fish parasites with notes on their distribution. *Journal of Fish Biology* **6**, 613–644. doi: [10.1111/j.1095-8649.1974.tb05104.x](https://doi.org/10.1111/j.1095-8649.1974.tb05104.x).

**Kennedy, C. R.** (1978). The parasite fauna of resident char *Salvelinus alpinus* from Arctic islands, with special reference to Bear Island. *Journal of Fish Biology* **13**, 457–466. doi: [10.1111/j.1095-8649.1978.tb03455.x](https://doi.org/10.1111/j.1095-8649.1978.tb03455.x).

**Knudsen, R., Amundsen, P.-A., Nilsen, R., Kristoffersen, R. and Klemetsen, A.** (2008). Food borne parasites as indicators of trophic segregation between Arctic charr and brown trout. *Environmental Biology of Fishes* **83**, 107–116. doi: [10.1007/s10641-007-9216-7](https://doi.org/10.1007/s10641-007-9216-7).

**Knudsen, R., Eloranta, A. P., Siwertsson, A., Paterson, R. A., Power, M. and Sandlund, O. T.** (2019). Introduction of Mysis relicta (Mysida) reduces niche segregation between deep-water Arctic charr morphs. *Hydrobiologia* **840**, 245–260. doi: [10.1007/s10750-019-3953-4](https://doi.org/10.1007/s10750-019-3953-4).

**Knudsen, R., Kristoffersen, R. and Amundsen, P.-A.** (1997). Parasite communities in two sympatric morphs of Arctic charr, *Salvelinus alpinus* (L.), in northern Norway. *Canadian Journal of Zoology* **75**, 2003–2009. doi: [10.1139/z97-833](https://doi.org/10.1139/z97-833).

**Kock, T. J., Perry, R. W., Hansen, G. S., Haner, P. V., Pope, A. C., Plumb, J. M., Cogliati, K. M. and Hansen, A. C.** (2019). *Juvenile Chinook salmon (Oncorhynchus tshawytscha) survival in Lookout Point Reservoir, Oregon, 2018*, 2331–1258. US Geological Survey.

**Koksvik, J. I.** (1994). Økologisk tilstandsrapport med hovedvekt på relasjoner mellom plankton og røye i Leksdalsvatn 1993.

**Kristmundsson, Á. and Richter, S. H.** Parasites of resident arctic charr, *Salvelinus alpinus*, and brown trout, *Salmo trutta*, in two lakes in Iceland.

**Kusterle, S., Halttunen, E., Thorstad, E. B., Næsje, T. F., Jensen, J. L. A., Gallo‐Bueno, A., Olague, E. and Rikardsen, A. H.** (2013). The gill maggot *Salmincola salmoneus* as an indicator of repeat spawning in Atlantic salmon *Salmo salar*. *Journal of Fish Biology* **82**, 1068–1073. doi: [10.1111/jfb.12040](https://doi.org/10.1111/jfb.12040).

**Kusterle, S., Kristoffersen, R. and Rikardsen, A.** (2012). Population dynamics of *Salmincola salmoneus* on Atlantic salmon in a northern Norwegian river. *Diseases of Aquatic Organisms* **100**, 59–70. doi: [10.3354/dao02489](https://doi.org/10.3354/dao02489).

**Lasee, B. A., Sutherland, D. R. and Moubry, M. E.** (1988). Host–parasite relationships between burbot ( *Lota lota* ) and adult *Salmincola lotae* (Copepoda). *Canadian Journal of Zoology* **66**, 2459–2463. doi: [10.1139/z88-364](https://doi.org/10.1139/z88-364).

**LeBlanc, R., MacMillan, J. L., Marcogliese, D. J. and Cone, D. K.** (2021). Parasitism of Brook Trout (*Salvelinus fontinalis*) during the first month post-emergence in streams in southwest Nova Scotia. *Proceedings of the Nova Scotian Institute of Science (NSIS)* **51**, 18. doi: [10.15273/pnsis.v51i2.11175](https://doi.org/10.15273/pnsis.v51i2.11175).

**Leong, T. S. and Holmes, J. C.** (1981). Communities of metazoan parasites in open water fishes of Cold Lake, Alberta. *Journal of Fish Biology* **18**, 693–713. doi: [10.1111/j.1095-8649.1981.tb03811.x](https://doi.org/10.1111/j.1095-8649.1981.tb03811.x).

**Lepak, J. M., Hansen, A. G., Hooten, M. B., Brauch, D. and Vigil, E. M.** (2022). Rapid proliferation of the parasitic copepod, *Salmincola californiensis* (Dana), on kokanee salmon, *Oncorhynchus nerka* (Walbaum), in a large Colorado reservoir. *Journal of Fish Diseases* **45**, 89–98. doi: [10.1111/jfd.13539](https://doi.org/10.1111/jfd.13539).

**Marcogliese, D. J. and Cone, D. K.** (1991). Do brook charr ( *Salvelinus fontinalis* ) from insular Newfoundland have different parasites than their mainland counterparts? *Canadian Journal of Zoology* **69**, 809–811. doi: [10.1139/z91-119](https://doi.org/10.1139/z91-119).

**McGladdery, S. E. and Johnston, C. E.** (1988). Egg development and control of the gill parasite, *Salmincola salmoneus*, on Atlantic salmon kelts (*Salmo salar*) exposed to four different regimes of temperature and photoperiod. *Aquaculture* **68**, 193–202. doi: [10.1016/0044-8486(88)90352-3](https://doi.org/10.1016/0044-8486(88)90352-3).

**Mendez, G. and Hill, M.** (2017). 2016 Juvenile Migration Test and Verification Study Annual Report. Available from: <https://public.crohms.org/tmt/documents/FPOM/2010/Willamette_Coordination/Steering_team/180501%20PRB%20Juv%20Mig%20Annual%20Report%202016.pdf>

**Meyer, M. C.** (1954). *The larger animal parasites of the fresh-water fishes of Maine*. Maine Department of Inland Fisheries and Game Augusta, ME, USA.

**Miller, R. B. and Kennedy, W.** (1948). Observations on the lake trout of Great Bear Lake. *Journal of the Fisheries Board of Canada* **7**, 176–189.

**Miller, R. B. and Kennedy, W.** (1948). Observations on the lake trout of Great Bear Lake. *Journal of the Fisheries Board of Canada* **7**, 176–189.

**Mitro, M. G., Lyons, J. D., Stewart, J. S., Cunningham, P. K. and Griffin, J. D. T.** (2019). Projected changes in Brook Trout and Brown Trout distribution in Wisconsin streams in the mid-twenty-first century in response to climate change. *Hydrobiologia* **840**, 215–226. doi: [10.1007/s10750-019-04020-3](https://doi.org/10.1007/s10750-019-04020-3).

**Mitro, M. G.** (2016). Brook Trout, Brown Trout, and Ectoparasitic Copepods *Salmincola edwardsii* : Species Interactions as a Proximate Cause of Brook Trout Loss Under Changing Environmental Conditions. *Transactions of the American Fisheries Society* **145**, 1223–1233. doi: [10.1080/00028487.2016.1219676](https://doi.org/10.1080/00028487.2016.1219676).

**Mitro, M. G. and Griffin, J. D.** (2018). Distribution, Prevalence, and Maximum Intensity of the Ectoparasitic Copepod *Salmincola* cf. *Edwardsii* in Brook Trout in Wisconsin Streams. *Journal of Parasitology* **104**, 628–638. doi: [10.1645/17-146](https://doi.org/10.1645/17-146).

**Mo, T. A., Appleby, C. and Sterud, E.** (1998). PARASITES OF GRAYLING (*THYMALLUS THYMALLUS*) FROM THE GLOMMA RIVER SYSTEM, SOUTH-EASTERN. *Bull Scand Soc Parasitol* **8**, 6–11.

**Modin, J. C. and Veek, T. M.** (2002). Biological Control of the Parasitic Copepod *Salmincola californiensis* in a Commercial Trout Hatchery on the Lower Merced River, California. *North American Journal of Aquaculture* **64**, 122–128. doi: [10.1577/1548-8454(2002)064<0122:BCOTPC>2.0.CO;2](https://doi.org/10.1577/1548-8454(2002)064%3C0122:BCOTPC%3E2.0.CO;2).

**Moles, A.** (1982) Parasite-Host Records of Alaskan Fishes. United States Department of Commerce, Washington, D. C., USA.

**Monod, T. and Vladykov, V.** (1931). Sur quelques copépodes parasites provenant de la Russie sous-carpathique (Tchécoslovaquie). *Annales de Parasitologie Humaine et Comparée* **9**, 202–224. doi: [10.1051/parasite/1931093202](https://doi.org/10.1051/parasite/1931093202).

**Monzyk, F. R., Friesen, T. A. and Romer, J. D.** (2015). Infection of Juvenile Salmonids by *Salmincola californiensis* (Copepoda: Lernaeopodidae) in Reservoirs and Streams of the Willamette River Basin, Oregon. *Transactions of the American Fisheries Society* **144**, 891–902. doi: [10.1080/00028487.2015.1052558](https://doi.org/10.1080/00028487.2015.1052558).

**Mudry, D. R. and McCart, P. J.** (1976). Metazoan Parasites of Arctic Char ( *Salvelinus alpinus* ) from the North Slope of Canada and Alaska. *Journal of the Fisheries Research Board of Canada* **33**, 271–275. doi: [10.1139/f76-037](https://doi.org/10.1139/f76-037).

**Mullin, B. R. and Reyda, F. B.** (2020). High Prevalence of the Copepod *Salmincola californiensis* in Steelhead Trout in Lake Ontario Following its Recent Invasion. *Journal of Parasitology* **106**, 198. doi: [10.1645/19-121](https://doi.org/10.1645/19-121).

**Murphy, C. A., Gerth, W. and Arismendi, I.** (2020). Hatching and survival of the salmon ‘gill maggot’ *Salmincola californiensis* (Copepoda: Lernaeopodidae) reveals thermal dependence and undocumented naupliar stage. *Parasitology* **147**, 1338–1343. doi: [10.1017/S0031182020001109](https://doi.org/10.1017/S0031182020001109).

**Muzzall, P. M.** (1986). Parasites of trout from the Au Sable River, Michigan, with emphasis on the population biology of *Cystidicoloides tenuissima*. *Canadian Journal of Zoology* **64**, 1549–1554. doi: [10.1139/z86-231](https://doi.org/10.1139/z86-231).

**Muzzall, P. M.** (2007). PARASITES OF JUVENILE BROOK TROUT (*SALVELINUS FONTINALIS*) FROM HUNT CREEK, MICHIGAN. *Journal of Parasitology* **93**, 313–317. doi: [10.1645/GE-3572.1](https://doi.org/10.1645/GE-3572.1).

**Muzzall, P. M. and Madenjian, C. P.** (2013). Parasites of Bloater *Coregonus hoyi* (Salmonidae) from Lake Michigan, U.S.A. *Comparative Parasitology* **80**, 164–170. doi: [10.1654/4617.1](https://doi.org/10.1654/4617.1).

**Nagasawa, K., Awakura, T. and Urawa, S.** (1989). A checklist and bibliography of parasites of freshwater fishes of Hokkaido. *Scientific Reports of the Hokkaido Fish Hatchery* **44**, 49.

**Nagasawa, K., Ikuta, K., Nakamura, H., Shikama, T. and Kitamura, S.** (1998). Occurrence and effects of the parasitic copepod *Salmincola carpionis* on salmonids in the Nikko District, central Japan. *Journal of marine systems* **15**, 269–272.

**Nagasawa, K., Ikuta, K. and Kitamura, S.** (1997). Distribution of *Salmincola carpionis* (Copepoda: Lernaeopodidae) in the Buccal Cavity. *Bull. Natl. Res. Inst. Aquacult. No* **26**, 35–39.

**Nagasawa, K., Kawanobe, M., Kumakawa, S., Matsuzawa, S. and Takehana, K.** (2018). Rediscovery of the salmonid parasite *Salmincola californiensis* (Crustacea: Copepoda: Lernaeopodidae) and identification of its host in the Ôtaki River, a tributary of the upper Kiso River, central Japan.

**Nagasawa, K., Urawa, S. and Awakura, T.** (1987). A checklist and bibliography of parasites of salmonids of Japan. *Hokkaido Salmon and Trout Hatchery Research Report,* 1–75.

**Nagasawa, K., Watanabe, J. R., Kimura, S. and Hara, A.** (1994). Infection of *Salmincola stellatu*s (Copepoda: Lernaeopodidae) on Sakhalin taimen *Hucho perryi* reared in Hokkaido. *Bull. Fac. Fish. Hokkaido Univ* **45**, 109–112.

**Nagasawa, K., Yamamoto, M., Sakurai, Y. and Kumagai, A.** (1995). Rediscovery in Japan and host association of *Salmincola carpionis* (Copepoda: Lernaeopodidae), a parasite of wild and reared freshwater salmonids. *Canadian Journal of Fisheries and Aquatic Sciences* **52**, 178–185. doi: [10.1139/f95-525](https://doi.org/10.1139/f95-525).

**Nagasawa, K., Yamamoto, M., Sakurai, Y. and Kumagai, A.** (1995). Rediscovery in Japan and host association of *Salmincola carpionis* (Copepoda: Lernaeopodidae), a parasite of wild and reared freshwater salmonids. *Canadian Journal of Fisheries and Aquatic Sciences* **52**, 178–185. doi: [10.1139/f95-525](https://doi.org/10.1139/f95-525).

**Nagasawa, K.** (2020). *Salmincola edwardsii* (Copepoda: Lernaeopodidae) parasitic on southern Asian Dolly Varden, *Salvelinus malma krascheninnikova*, from Hokkaido Island, Japan, with the southernmost distribution record of the copepod in Asia. Species Diversity 25 197–203, doi: 10.12782/specdiv.25.197.

**Nagasawa, K.** (2020). *Salmincola markewitschi* (Copepoda: Lernaeopodidae) Parasitic on Whitespotted Char, *Salvelinus leucomaenis*, in a Mountain Stream of Honshu Island, Central Japan. *Species Diversity* **25**, 369–375. doi: [10.12782/specdiv.25.369](https://doi.org/10.12782/specdiv.25.369).

**Nagasawa, K.** (2021). Two Copepods *Salmincola edwardsii* and *Salmincola markewitschi* (Lernaeopodidae) Parasitic on Chars (*Salvelinus* spp.) Reared in a Salmon Museum, Northern Japan. *Species Diversity* **26**, 137–143. doi: [10.12782/specdiv.26.137](https://doi.org/10.12782/specdiv.26.137).

**Nagasawa, K.** (2021a). Gill lesions caused by the parasitic copepod Salmincola edwardsii in southern Asian Dolly Varden, *Salvelinus malma krascheninnikova*, from Hokkaido Island, Japan. *Nature of Kagoshima* **47**, 121–124.

**Nagasawa, K.** (2021b). Two Copepods *Salmincola edwardsii* and *Salmincola markewitschi* (Lernaeopodidae) Parasitic on Chars (*Salvelinus* spp.) Reared in a Salmon Museum, Northern Japan. *Species Diversity* **26**, 137–143. doi: [10.12782/specdiv.26.137](https://doi.org/10.12782/specdiv.26.137).

**Nagasawa, K. and Ishiyama, N.** (2021). *Salmincola markewitschi* (Copepoda: Lernaeopididae), a parasite of whitespotted charr, *Salvelinus leucomaenis*, from Ishikawa Prefecture, central Japan. *TAXA, Proc. Japan. Soc. Syst. Zool* **50**, 11–19.

**Nagasawa, K. and Sakaki, M.** Infection of *Salmincola carpionis* (Copepoda: Lernaeopodidae) on whitespotted charr, *Salvelinus leucomaenis* (Salmonidae), reared in northern Honshu, Japan. **46**, 113-115.

**Nagasawa, K. and Urawa, S.** (1991). New records of the parasitic copepod *Salmincola stellatus* from Sakhalin taimen (Hucho perryi) in Hokkaido, with a note on its attachment site. 北海道さけ・ますふ化場研究報告 57–59.

**Nagasawa, K. and Urawa, S.** (2002). Infection of *Salmincola californiensis* (Copepoda: Lernaeopodidae) on Juvenile Masu Salmon (*Oncorhynchus masou*) from a Stream in Hokkaido. Bulletin of the National Salmon Resources Center, **5**, 7-12.

**Nagasawa, K. and Urawa, S.** (2022). Occurrence of *Salmincola edwardsii* (Olsson, 1869) and *Salmincola markewitschi* Shedko & Shedko, 2002 (Copepoda: Lernaeopodidae) on stream-dwelling salmonids in eastern Hokkaido, Japan, with observations on the morphology of the copepods. *Crustacean Research* **51**, 91–101. doi: [10.18353/crustacea.51.0_91](https://doi.org/10.18353/crustacea.51.0_91).

**Neal, T., Kent, M. L., Sanders, J., Schreck, C. B. and Peterson, J. T.** (2021). Laboratory infection rates and associated mortality of juvenile Chinook Salmon ( *Oncorhynchus tshawytscha* ) from parasitic copepod ( *Salmincola californiensis* ). *Journal of Fish Diseases* **44**, 1423–1434. doi: [10.1111/jfd.13450](https://doi.org/10.1111/jfd.13450).

**Nikulina, Y. S. and Polyaeva, K. V.** (2020). Morphology, biology and parasite fauna of the least cisco (*Coregonus sardinella*) of the Yenisei River. *Biosystems Diversity* **28**, 230–237. doi: [10.15421/012030](https://doi.org/10.15421/012030).

**Olk, T. R., Henriksen, A.-C., Dolven, S. I., Haukø, M. L., Lydersen, E. and Mo, T. A.** (2020). Factors determining parasite abundance in European perch, Perca fluviatilis, European whitefish, *Coregonus lavaretus*, and Arctic charr, *Salvelinus alpinus*, in an oligotrophic lake, southern Norway. *Fauna norvegica* **40**, 109–129. doi: [10.5324/fn.v40i0.3444](https://doi.org/10.5324/fn.v40i0.3444).

**O’Riordan, C.** (1964). Parasitic copepods in the collections of the National Museum of Ireland.pp. 371–378. JSTOR.

**Østbye, K., Næsje, T. F., Bernatchez, L., Sandlund, O. T. and Hindar, K.** (2005). Morphological divergence and origin of sympatric populations of European whitefish ( *Coregonus lavaretus* L.) in Lake Femund, Norway. *Journal of Evolutionary Biology* **18**, 683–702. doi: [10.1111/j.1420-9101.2004.00844.x](https://doi.org/10.1111/j.1420-9101.2004.00844.x).

**Parker, H. H.** (1996). Population ecology of landlocked Arctic charr, *Salvelinus alpinus L*., in the Canadian High Arctic. doi: [10.21954/OU.RO.0000E0D8](https://doi.org/10.21954/OU.RO.0000E0D8).

**Partington, J. D. and Mills, C. A.** (1988). An electrophoretic and biometric study of Arctic charr, *Salvelinm alpinus* (L.), from ten British lakes. *Journal of Fish Biology* **33**, 791–814. doi: [10.1111/j.1095-8649.1988.tb05524.x](https://doi.org/10.1111/j.1095-8649.1988.tb05524.x).

**Paterson, R. A., Knudsen, R., Blasco-Costa, I., Dunn, A. M., Hytterød, S. and Hansen, H.** (2019b). Determinants of parasite distribution in Arctic charr populations: catchment structure versus dispersal potential. *Journal of Helminthology* **93**, 559–566. doi: [10.1017/S0022149X18000482](https://doi.org/10.1017/S0022149X18000482).

**Paterson, R. A., Nefjodova, J., Salis, R. K. and Knudsen, R.** (2019a). Exploring trophic niches and parasite communities of sympatric Arctic charr and brown trout populations of southern Norway. *Hydrobiologia* **840**, 271–280. doi: [10.1007/s10750-019-3956-1](https://doi.org/10.1007/s10750-019-3956-1).

**Pawaputanon, K.** (1980). Effects of parasitic copepod, *Salmincola californiensis* (Dana, 1852) on juvenile sockeye salmon, *Oncorhynchus nerka* (Walbaum). (Doctoral dissertation) University of British Columbia. Vancouver, British Columbia, Canada. Available from: <https://open.library.ubc.ca/media/download/pdf/831/1.0095204/1>

**Pellerin, J. and Grondin, J.** (1998). Assessing the State of Arctic Ecosystem Health: Bridging Inuit Viewpoints and Biological Endpoints on Fish Health. *Ecosystem Health* **4**, 236–247. doi: [10.1046/j.1526-0992.1998.98099.x](https://doi.org/10.1046/j.1526-0992.1998.98099.x).

**Pietrock, M. and Hursky, O.** (2011). Fish and ecosystem health as determined by parasite communities of lake whitefish (*Coregonus clupeaformis*) from Saskatchewan boreal lakes. *Water Quality Research Journal* **46**, 219–229. doi: [10.2166/wqrjc.2011.004](https://doi.org/10.2166/wqrjc.2011.004).

**Pippy, J. H.** (1969). Preliminary report on parasites as biological tags in Atlantic salmon (*Salmo salar*). I. Investigations 1966 to 1968. Technical Report of the Fisheries Research Board of Canada. **134.**

**Pope, A. C., Kock, T. J., Perry, R. W., Cogliati, K. M., O’Malley, K. G., Murphy, C. A., Hance, D. J. and Fielding, S. D.** (2024). Using parentage-based tagging to estimate survival of Chinook salmon fry in a large storage reservoir. *Environmental Biology of Fishes* **107**, 735–754. doi: [10.1007/s10641-024-01564-9](https://doi.org/10.1007/s10641-024-01564-9).

**Pospekhov, V., Atrashkevich, G. and Orlovskaya, O.** (2010). Fish parasites from the Gizhiga River basin(northern coast of the Okhotsk sea). *Izvestiya Tikhookeanskogo nauchno-issledovatel’skogo rybokhozyajstvennogo tsentra* **163**,.

**Pospekhov, V. V., Atrashkevich, G. I. and Orlovskaya, O. M.** (2020). Parasites of graylings (Thymallidae: *Thymallus*) from the northern continental coast of the Okhotsk Sea. *Izvestiya TINRO* **200**, 965–977. doi: [10.26428/1606-9919-2020-200-965-977](https://doi.org/10.26428/1606-9919-2020-200-965-977).

**Poulin, R., Conley, D. C. and Curtis, M. A.** (1990b). Effects of temperature fluctuations and photoperiod on hatching in the parasitic copepod *Salmincola edwardsii*. *Canadian Journal of Zoology* **68**, 1330–1332. doi: [10.1139/z90-199](https://doi.org/10.1139/z90-199).

**Poulin, R., Curtis, M. A. and Rau, M. E.** (1990a). Responses of the fish ectoparasite *Salmincola edwardsii* (Copepoda) to stimulation, and their implication for host-finding. *Parasitology* **100**, 417–421. doi: [10.1017/S0031182000078707](https://doi.org/10.1017/S0031182000078707).

**Poulin, R., Curtis, M. A. and Rau, M. E.** (1991b). Size, Behaviour, and Acquisition of Ectoparasitic Copepods by Brook Trout, *Salvelinus fontinalis*. *Oikos* **61**, 169. doi: [10.2307/3545334](https://doi.org/10.2307/3545334).

**Poulin, R., Rau, M. E. and Curtis, M. A.** (1991a). Infection of brook trout fry, *Salvelinus fontinalis*, by ectoparasitic copepods: the role of host behaviour and initial parasite load. *Animal Behaviour* **41**, 467–476. doi: [10.1016/S0003-3472(05)80849-8](https://doi.org/10.1016/S0003-3472(05)80849-8).

**Pugachev, O.**(1980). Генезис паразитофауны лососевых рыб Евразии. *Паразитология* **14**, 403–410.

**Radziun, K.** (1993). Observations on the Arctic charr, *Salvelinus alpinus* (L., 1758) from the Hornsund region (Vest Spitsbergen) in 1985-1987. *Acta Ichthyologica et Piscatoria* **23**, 7–22.

**Rawson, D. S.** (1951). Studies of the Fish of Great Slave Lake. *Journal of the Fisheries Research Board of Canada* **8b**, 207–240. doi: [10.1139/f50-014](https://doi.org/10.1139/f50-014).

**Reeves, W. K. (2015).** Checklist of copepods (Crustacea: Calanoida, Cyclopoida, Harpacticoida) from Wyoming, USA, with new state records. *Check List* 11, 1764. doi: [10.15560/11.5.1764](https://doi.org/10.15560/11.5.1764).

**Refsnes, B.,** (2014). Parallelism in parasite infections in two populations of polymorphic Arctic charr (*Salvelinus alpinus* L.) in northern Norway (Master Thesis) The Arctic University of Norway, Tromsø, Norway. Available at: <https://munin.uit.no/handle/10037/6551>

**Ricker, W.** (1938). “ Residual” and kokanee salmon in Cultus Lake. *Journal of the Fisheries Board of Canada* **4**, 192–218.

**Riis, J. C.** Parasites of Salmonid Fishes from Southcentral Alaska. (Master thesis). South Dakota State University, Brookings, South Dakota, USA. available at: <https://openprairie.sdstate.edu/cgi/viewcontent.cgi?article=5646&context=etd>

**Roberts, R. J., Johnson, K. A. and Casten, M. T.** (2004). Control of *Salmincola californiensis* (Copepoda: Lernaeapodidae) in rainbow trout, *Oncorhynchus mykiss* (Walbaum): a clinical and histopathological study. *Journal of Fish Diseases* **27**, 73–79. doi: [10.1046/j.1365-2761.2003.00508.x](https://doi.org/10.1046/j.1365-2761.2003.00508.x).

**Rokicki, J.** (1986). Parasitic Crustacea of marine fishe*s. Wiadomości Parazytologiczne* **32**, 493–496.

**Rokicki, J.** (1991). Parasitic Crustacea of marine fishes. *Wiadomości Parazytologiczne* **37**, 137–140.

**Roon, S.R.** (2014) Distribution and coinfection of microparasites and macroparasites in juvenile salmonids in three upper Willamette River tributaries. (Master thesis). Oregon State University, Corvallis, Oregon, USA. Available at: <https://ir.library.oregonstate.edu/downloads/8623j111s>

**Rubinoff, D.** (2006). Utility of Mitochondrial DNA Barcodes in Species Conservation. *Conservation Biology* **20**, 1026–1033. doi: [10.1111/j.1523-1739.2006.00372.x](https://doi.org/10.1111/j.1523-1739.2006.00372.x).

**Ruiz, C. F., Rash, J. M., Besler, D. A., Roberts, J. R., Warren, M. B., Arias, C. R. and Bullard, S. A.** (2017). Exotic “Gill Lice” Species (Copepoda: Lernaeopodidae: *Salmincola* SPP.) Infect Rainbow Trout ( *Oncorhynchus mykiss* ) and Brook Trout ( *Salvelinus fontinalis* ) in the Southeastern United States. *Journal of Parasitology* **103**, 377–389. doi: [10.1645/16-165](https://doi.org/10.1645/16-165).

**Rullestad, I.** (2021). Parasites Found in Pink Salmon (*Oncorhynchus gorbuscha*) Caught in the Feeding Areas in the Norwegian Sea.

**Sandeman, I. and Pippy, J.** (1967). Parasites of freshwater fishes (Salmonidae and Coregonidae) of insular Newfoundland. *Journal of the Fisheries Board of Canada* **24**, 1911–1943.

**Savage, J.** (1935). Copepod Infection of Speckled Trout. *Transactions of the American Fisheries Society* **65**, 334–339. doi: [10.1577/1548-8659(1935)65[334:CIOST]2.0.CO;2](https://doi.org/10.1577/1548-8659(1935)65%5B334:CIOST%5D2.0.CO;2).

**Seidlová, L., Benovics, M. and Šimková, A.** (2022). Gill monogeneans of neotropical cichlid fish: diversity, phylogenetic relationships, and host-parasite cophylogenetic associations. *International Journal for Parasitology* **52**, 603–615. doi: [10.1016/j.ijpara.2022.05.001](https://doi.org/10.1016/j.ijpara.2022.05.001).

**Shedko, M.** (2004). New species of parasitic copepod *Salmincola* mica sp. n.(Lernaeopodidae) from the round whitefish *Prosopium cylindraceum* (Coregonidae), of the Anadyr River. *Vestnik zoologii,* **38**, 39-45.

**Shedko, M. B., & Shedko, S. V.** (2002). Parasitic copepods of the genus *Salmincola* (Lernaeopodidae) from the far eastern chars *Salvelinus* (Salmonidae) with description of the new species *S. markewitschi*. *Zoologicheskii Zhurnal* **81,** 141-153

**Shedko, M. B., & Shedko, S. V.** (2003). МОРФОЛОГИЯ И РАСПРОСТРАНЕНИЕ *SALMINCOLA* STELLATUS (COPEPODA: LERNAEOPODIDAE) ОТ САХАЛИНСКОГО ТАЙМЕНЯ PARAHUCHO PERRYI (SALMONIDAE) ИЗ ПРИМОРЬЯ. *Паразитология* **37**, 60–68.

**Shedko, S. V., Shedko, M. B., Miroshnichenko, I. L. and Nemkova, G. A.** (2023). DNA Identification of Parasitic Copepods *Salmincola* (Copepoda, Siphonostomatoida, Lernaeopodidae): Variability and Rate of Evolution of the Mitochondrial Cytochrome c Oxidase Subunit I Gene. *Russian Journal of Genetics* **59**, 1022–1031. doi: [10.1134/S1022795423100113](https://doi.org/10.1134/S1022795423100113).

**Shesterikov, D., Dugarov, Z. N., Burdukovskaya, T., Tolochko, L., Zhepkholova, О., Sondueva, L., Baldanova, D., Khamnueva, T., Vokin, A. and Batueva, M.-D.** (2019). Parasite Fauna of White Baikal Grayling *Thymallus brevipinnis* Svetovidov, 1931 in the Bezymiannaia Bay, Lake Baikal (Republic of Buryatia, Russia). *The bulletin of Irkutsk State University.«Geoarchaeology, Ethnology, and Anthropology Series»* 87–97.

**Sobecka, E. and Piasecki, W.** (1993). Parastic fauna of Arctic charr, *Salvelinus alpinus* (L., l758) from the Hornsund region (Spitsbergen). *Acta Ichthyologica et Piscatoria* **23**, 99–106.

**Stables, T. and Perrin, C.** (2014). Abundance and biomass of fish in Stave Reservoir in fall 2013. *Draft report prepared by Limnotek Research and Development Inc. and Shuksan Fisheries Consulting for BC Hydro*. Available from: <https://www.bchydro.com/content/dam/BCHydro/customer-portal/documents/corporate/environment-sustainability/water-use-planning/lower-mainland/sflmon-3-yr9-2014-02-01.pdf>

**Stańkowska-Radziun, M. and Radziun, K.** (1993). Observations on the development of *Salmincola edwardsii* (Olsson, 1869)(Copepoda: Lernaeopodidae) parasitizing the Arctic charr (*Salvelinus alpinus* (L.)) in the Hornsund region (Vest Spitsbergen). *Acta Ichthyologica et Piscatoria* **23**, 107–114.

**Suchomel, A. D. and Billman, E. J.** (2021). Prevalence and Intensity of Infection of the Parasitic Copepod *Salmincola californiensis* on Rainbow Trout in Birch Creek, Idaho. *Western North American Naturalist* **81**, doi: [10.3398/064.081.0413](https://doi.org/10.3398/064.081.0413).

**Sukhanova, L. V., Smirnov, V. V., Smirnova-Zalumi, N. S., Belomestnykh, T. V. and Kirilchik, S. V.** (2012). Molecular Phylogeography of Lake Baikal Coregonid Fishes. *Advances in Limnology* **63**, 261–283. doi: [10.1127/advlim/63/2012/261](https://doi.org/10.1127/advlim/63/2012/261).

**Sutherland, D. R. and Wittrock, D. D.** (1985). The effects of *Salmincola californiensis* (Copepoda: Lernaeopodidae) on the gills of farm-raised rainbow trout, *Salmo gairdneri*. *Canadian Journal of Zoology* **63**, 2893–2901. doi: [10.1139/z85-433](https://doi.org/10.1139/z85-433).

**Swain‐Menzel, H. N., & Billman, E. J.,** (2023). Low‐level infection of parasitic copepods on Rainbow Trout does not affect vulnerability to angling or short‐term survival following catch‐and‐release angling. *North American Journal of Fisheries Management* **43**, 1789-1798

**Taylor, E. B.** (2016). The Arctic char (*Salvelinus alpinus*) “complex” in North America revisited. *Hydrobiologia* **783**, 283–293. doi: [10.1007/s10750-015-2613-6](https://doi.org/10.1007/s10750-015-2613-6).

**Thiede, G. P., Kern, J. C., Weldon, M. K., Dale, A. R., Thiesfeld, S. and Buckman, M.** (2002). Lake Billy Chinook sockeye salmon and kokanee research study 1996–2000. *Draft Project Completion Report. Pelton-Round Butte Hydroelectric Project. For Portland General Electric Company*.

**Tidd, W. M.** A List of Parasitic Copepods and Their Fish Hosts from Lake Erie. *The Ohio journal of science.* **6**, 453-454.

**Tidd, W. M. and Bangham, R. V.** (1945). A copepod parasite of the cisco from Trout Lake, Wisconsin. Available from: <https://kb.osu.edu/bitstreams/34c881ef-1d7f-5bb1-968e-48dd7aca01a9/download>

**Vaughan, G. E. and Coble, D. W.** (1975). Sublethal effects of three ectoparasites on fish. *Journal of Fish Biology* **7**, 283–294. doi: [10.1111/j.1095-8649.1975.tb04601.x](https://doi.org/10.1111/j.1095-8649.1975.tb04601.x).

**Watson, R. A.** (1977). Metazoan parasites from whitefish, cisco and pike from Southern Indian Lake, Manitoba: a preimpoundment and diversion analysis. (Master thesis). University of Manitoba, Winnipeg, Manitoba, Canada. Available from: <https://mspace.lib.umanitoba.ca/bitstream/handle/1993/6346/Watson_Metazoan_parasites.pdf>

**West, R. L.** (1986). *Baseline histopathological and contaminant studies of four Arctic fish species in Beaufort Lagoon, Arctic National Wildlife Refuge, Alaska*. Fairbanks Fishery Resources Station.

**White, C. F. H., Gray, M. A., Kidd, K. A., Duffy, M. S., Lento, J. and Monk, W. A.** (2020). Prevalence and Intensity of *Salmincola edwardsii* in Brook Trout in Northwest New Brunswick, Canada. *Journal of Aquatic Animal Health* **32**, 11–20. doi: [10.1002/aah.10091](https://doi.org/10.1002/aah.10091).

**White, C. F. H.** (2020). Fish health effects from forest harvest and ectoparasitic copepods in northern New Brunswick. (Master thesis). University of New Brunswick, Fredericton, New Brunswick, Canada. Available from: <https://unbscholar.lib.unb.ca/bitstreams/925084f2-c28f-4f59-b12b-c956afa2860e/download>

**Wiles, M. and May, A.** (1968). *Biology and fishery of the West Newfoundland cod stock*. International Commission for the Northwest Atlantic Fisheries.

**Wilson, C. B.** (1915). *North American parasitic copepods belonging to the Lernaeopodidae: With a revision of the entire family*. US Government Printing Office, Washington, D.C., USA.

**Yau, M. M. and Taylor, E. B.** (2013). Environmental and anthropogenic correlates of hybridization between westslope cutthroat trout (*Oncorhynchus clarkii lewisi*) and introduced rainbow trout (*O. mykis*s). *Conservation Genetics* **14**, 885–900. doi: [10.1007/s10592-013-0485-8](https://doi.org/10.1007/s10592-013-0485-8).
